# Transcriptomic determinants of the response of ST-111 *Pseudomonas aeruginosa* AG1 to ciprofloxacin identified by a top-down systems biology approach

**DOI:** 10.1038/s41598-020-70581-2

**Published:** 2020-08-13

**Authors:** José Arturo Molina-Mora, Diana Chinchilla-Montero, Maribel Chavarría-Azofeifa, Alejandro J. Ulloa-Morales, Rebeca Campos-Sánchez, Rodrigo Mora-Rodríguez, Leming Shi, Fernando García

**Affiliations:** 1 Centro de Investigación en Enfermedades Tropicales (CIET), Facultad de Microbiología, Universidad de Costa Rica, San José, Costa Rica; 2 Chemical Genomics Centre (CGC), Max-Planck-Institute for Molecular Physiology, Dortmund, Germany; 3 Centro de Investigación en Biología Celular Y Molecular (CIBCM), Facultad de Microbiología, Universidad de Costa Rica, San José, Costa Rica; 4 Human Phenome Institute (HuPI), Fudan University, Shanghai, China

**Keywords:** Cellular signalling networks, Data processing, Gene regulatory networks, Network topology, Systems biology, Antimicrobials, Bacteria, Bacteriology, Bacteriophages, Microbial genetics

## Abstract

*Pseudomonas aeruginosa* is an opportunistic pathogen that thrives in diverse environments and causes a variety of human infections. *Pseudomonas aeruginosa* AG1 (PaeAG1) is a high-risk sequence type 111 (ST-111) strain isolated from a Costa Rican hospital in 2010. PaeAG1 has both blaVIM-2 and blaIMP-18 genes encoding for metallo-β-lactamases, and it is resistant to β-lactams (including carbapenems), aminoglycosides, and fluoroquinolones. Ciprofloxacin (CIP) is an antibiotic commonly used to treat *P. aeruginosa* infections, and it is known to produce DNA damage, triggering a complex molecular response. In order to evaluate the effects of a sub-inhibitory CIP concentration on PaeAG1, growth curves using increasing CIP concentrations were compared. We then measured gene expression using RNA-Seq at three time points (0, 2.5 and 5 h) after CIP exposure to identify the transcriptomic determinants of the response (i.e. hub genes, gene clusters and enriched pathways). Changes in expression were determined using differential expression analysis and network analysis using a top–down systems biology approach. A hybrid model using database-based and co-expression analysis approaches was implemented to predict gene–gene interactions. We observed a reduction of the growth curve rate as the sub-inhibitory CIP concentrations were increased. In the transcriptomic analysis, we detected that over time CIP treatment resulted in the differential expression of 518 genes, showing a complex impact at the molecular level. The transcriptomic determinants were 14 hub genes, multiple gene clusters at different levels (associated to hub genes or as co-expression modules) and 15 enriched pathways. Down-regulation of genes implicated in several metabolism pathways, virulence elements and ribosomal activity was observed. In contrast, amino acid catabolism, RpoS factor, proteases, and phenazines genes were up-regulated. Remarkably, > 80 resident-phage genes were up-regulated after CIP treatment, which was validated at phenomic level using a phage plaque assay. Thus, reduction of the growth curve rate and increasing phage induction was evidenced as the CIP concentrations were increased. In summary, transcriptomic and network analyses, as well as the growth curves and phage plaque assays provide evidence that PaeAG1 presents a complex, concentration-dependent response to sub-inhibitory CIP exposure, showing pleiotropic effects at the systems level. Manipulation of these determinants, such as phage genes, could be used to gain more insights about the regulation of responses in PaeAG1 as well as the identification of possible therapeutic targets. To our knowledge, this is the first report of the transcriptomic analysis of CIP response in a ST-111 high-risk *P. aeruginosa* strain, in particular using a top-down systems biology approach.

## Introduction

*Pseudomonas aeruginosa* is a ubiquitous Gram-negative organism which thrives in diverse environments and acts as an opportunistic pathogen^1^. The ability of this pathogen to cause a variety of human infections is facilitated by its nutritional versatility^2^, resistance to a wide spectrum of antibiotics, and virulence factors^3,4^. *Pseudomonas aeruginosa* AG1 (PaeAG1) is a multiresistant high-risk sequence type 111 (ST-111) strain (GenBank CP045739)^5^. It was isolated from a Costa Rican hospital and it was the first report of an isolate of *P. aeruginosa* carrying both blaVIM-2 and blaIMP-18 genes encoding for metallo-β-lactamases enzymes (carbapenemases), located in two independent integrons^5,6^. PaeAG1 is resistant to β-lactams (including carbapenems), aminoglycosides, and fluoroquinolones, being only sensitive to colistin. In addition to this multidrug-resistant feature, as in other *P. aeruginosa* strains, the ability to colonize nosocomial environments makes this strain a high-risk clone^7^. Owed to this antibiotic resistance profile, including resistance to carbapenems, PaeAG1 is classified as a Priority 1 (critical) organism according to the World Health Organization (WHO)^8^.

Antibiotic resistance is a major threat to public health because it compromises the administration of appropriate antibiotic therapy, and reduces the therapeutic options to treat infections, increasing patient morbidity and mortality^9,10^. This situation is aggravated by the emergence of strains resistant to multiple antibiotics^11^, limitation of the knowledge of interactions with pathogens and mechanisms of action of antimicrobial agents, and development of new antibiotics^12^. Use of antibiotics below the minimum inhibitory concentration (MIC) or sub-inhibitory concentrations also contributes to antibiotic resistance as they allow strains to continue growing and can select for pre-existing resistant organisms^13^. Since sub-inhibitory antibiotic concentrations are found in many natural environments, bacteria can naturally trigger mechanisms of tolerance^14^. However, the fundamental mechanisms of bacterial tolerance to antibiotics have not been fully elucidated^15^.

It has been shown that the perturbation induced by many antibiotics leads to stress conditions in prokaryotic cells^16^, which can induce DNA damage^17^. Stressors activate the regulation of gene expression or the activity and stability of existing proteins to induce adaptation mechanisms^16^. Organisms have evolved numerous DNA repair pathways to eliminate DNA damage and restart DNA replication^18^. Regulatory networks of transcriptional responses to DNA damage involves not only DNA repair enzymes, but also diverse proteins with roles in cell division, metabolism modulation, genetic rearrangements and exchange, mutation, and virulence factor production^19^.

Ciprofloxacin (CIP) is a fluoroquinolone antibiotic used to treat *P. aeruginosa* infections^20^. CIP is well-known to produce DNA damage by inhibiting DNA gyrase and topoisomerase IV, leading to DNA strand breaks^21^. Mutations in these genes are responsible for CIP resistance by losing drug affinity^22^. CIP has been used to study stress responses in this bacterial group^12,23^, in particular with the induction of the SOS response as a mechanism of DNA damage repair^17,24,25^. In *P. aeruginosa,* the SOS response regulon is composed of 15 genes, including *recA* and *lexA* genes^26^. Upon DNA damage, RecA recognizes the single-stranded DNA (ssDNA) forming filaments and induces the autocleavage of the repressor LexA. This response leads to the expression of genes related to DNA damage repair^27^. Other LexA-like repressors are regulated during SOS activation, including elements of phages and pyocines^19^. SOS also mediates responses to resistance element transfer, generation of mutations and evolution of resistance^26^, as well as appearance of persister cells^24^.

However, modulation of stress responses after DNA damage is not limited to SOS response. RpoS is a general stress sigma factor (σS) known as a central element in a regulatory network that governs the expression of stationary-phase-induced genes^28^ to maintain cell viability^29^. This regulator is strongly induced when cells are exposed to various stress conditions, including antibiotics, pH downshift, starvation, and hyperosmolarity^30^. RpoS regulates more than 50 genes in *Pseudomonas aeruginosa*^31^*,* including virulence factors^32^.

The SOS and RpoS regulons are complementary mechanisms in response to certain stresses and that protect bacteria from DNA damage^33^. Lon protease^11^ and AmpR^34^ can modulate both SOS and RpoS regulons. In addition, both responses can regulate key genes such as *polB*^18^, *iraD*^19^, and *dinB*^33^. The connection between RpoS and SOS responses seems to be associated with a mechanism to maximize survival and fitness of cells, and to maintain genome stability^18^. These responses can modulate virulence factors (including quorum sensing and biofilm formation), and increase homologous recombination and mutation frequencies^33,35^. However, other SOS and RpoS independent mechanisms are also known to be present in bacteria^36^, including *P. aeruginosa* after CIP treatment^12,26^ with variable results depending on strains and showing a mosaic response^12^*.*

Although the full mechanisms of all these molecular responses are not well understood, it is known that cells respond to stress conditions by complex regulatory systems that control gene expression^37^. Since a key objective in biological research is to describe molecular interactions^38^, the use of networks analysis is a common approach to describe complex biological systems and to mathematically model gene–gene interactions (GGI) with graphical representations (genes as nodes and interactions as edges)^39^. Molecules are thereby studied not only at a single level, but emergent properties are identified to describe and understand the complexity of the gene networking response and the emergent properties towards the stress condition. Functional status of genes by a top-down systems biology perspective, starting from “whole”-omics data to identify specific determinants or elements of biological importance, can be evaluated by construction of large scale networks^40^. For this purpose, data analysis from high-throughput technologies such as microarrays and RNA sequencing (RNA-Seq) can be used to describe molecular interactions at transcriptomic level^38,41^. Thus, to understand or to infer mechanisms associated with the transcriptional response, it is possible to build gene regulatory networks either using databases or based on co-expression data^39,42,43^. These networks allow to gain insight into response to stress conditions^44^, leading to the identification of gene clusters or even hub genes as candidate biomarkers or modulators with potential to become key therapeutic targets^43,45^.

In *P. aeruginosa*, rapid adaptation to stress conditions is partially explained by the modulation of the global gene expression, which represents around 8% of all coding genes^3^. This regulation induces pleiotropic effects on its genomic regulatory network^46^, as previously shown using systems biology^47^, and the transcriptomic profiling of the response to CIP^12,26,48^.

In this work we first evaluated PaeAG1 growth at sub-inhibitory CIP concentrations, showing growth reduction as CIP was increased. We hypothesized that after exposing PaeAG1 to ciprofloxacin, even at sub-inhibitory concentrations, transcriptomic determinants will be triggered, including bacterial growth modulators. Thus, the aim was to identify transcriptomic determinants associated with the response to CIP in PaeAG1 using RNA-Seq profiling and network analysis by a top-down systems biology approach. Results showed that PaeAG1 generates a complex response to CIP exposure, evidencing pleiotropic effects involving the regulation of multiple hub genes, gene clusters and enriched pathways (transcriptomic determinants), many of them related to growth. As evidenced at the transcriptomic and the phenomic levels, phage induction was a particular trait modulated by CIP in a concentration-dependent manner with a correlation with bacterial growth reduction.

## Methods

**Figure 1:**
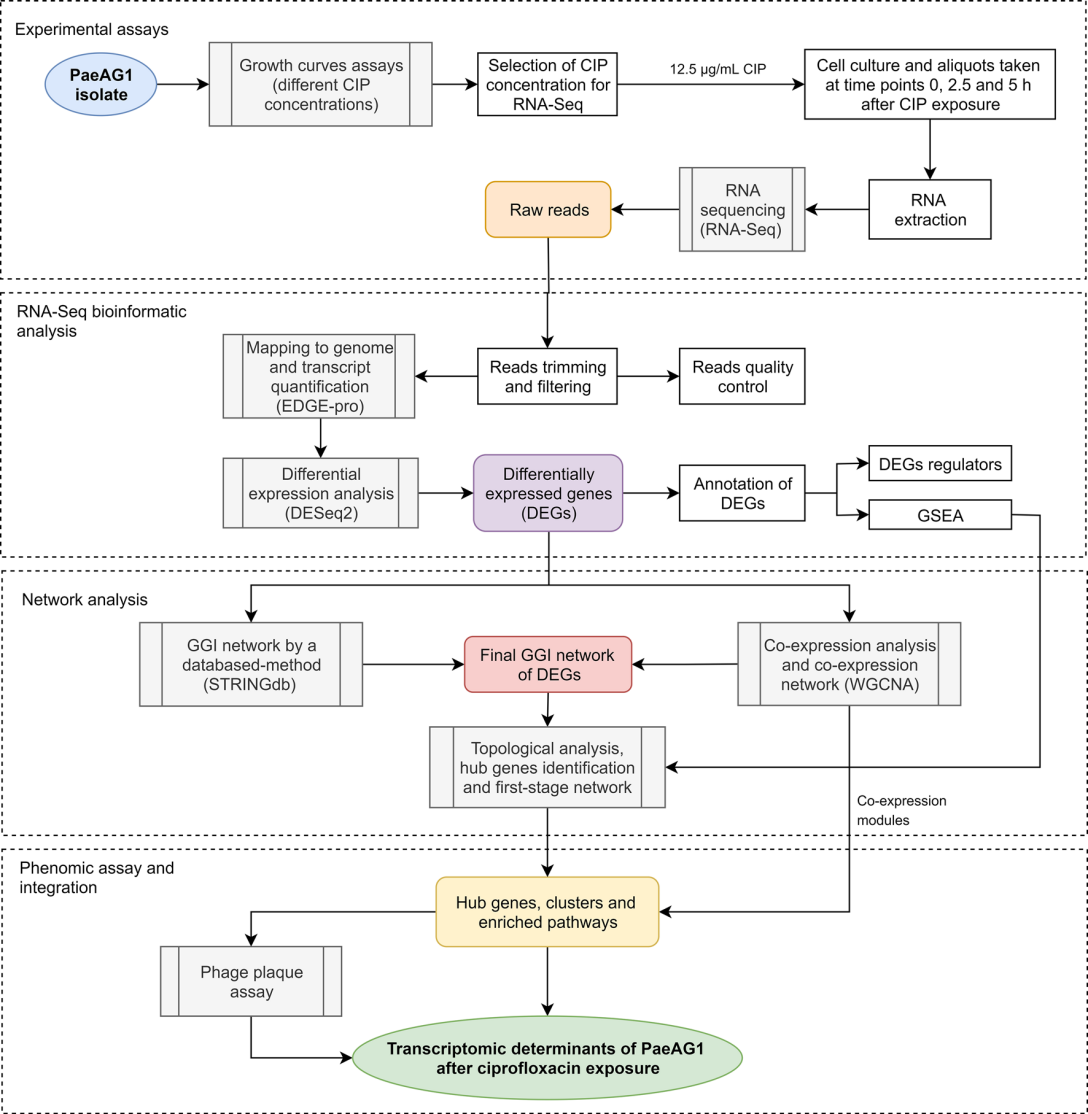
General pipeline to identify the transcriptomic determinants of the response of *P. aeruginosa* AG1 to ciprofloxacin (CIP). After growth curves assessment, a specific CIP concentration was used to sequence RNA (RNA-Seq) at 0, 2.5 and 5 h after exposure. DEGs were identified and used to build GGI networks. Transcriptomic determinants were identified by network analysis. Findings were verified at phenomic level using a phage plaque assay.

The general pipeline followed in this study to identify the transcriptomic determinants associated with the response to CIP in PaeAG1 is shown in Fig. 1.

### Bacterial isolate

The PaeAG1 strain is a Costa Rican multiresistant isolate from a sputum sample of a patient with pneumonia at the Intensive Care Unit of the San Juan de Dios Hospital (San José, Costa Rica)^6^. PaeAG1 exhibits resistance to β-lactams (including carbapenems, MIC_Meropenem_ 32 µg/mL and MIC_Imipenem_ > 32 µg/mL), aminoglycosides (MIC_Gentamycin_ 128 µg/mL and MIC_Tobramycin_ > 192 µg/mL) and fluoroquinolones (MIC_Ciprofloxacin_ 32 µg/mL), and it is only sensitive to colistin (MIC_Colistin_ 2 µg/mL). We recently assembled and annotated the PaeAG1 genome^5^, and genome sequence and annotation are available in Genbank under accession CP045739 (Bioproject PRJNA587210).

### Growth curves assay

Three independent cultures of PaeAG1 cells were grown to exponential-phase overnight in Lysogenic Broth (LB) at 37 °C with shaking (pre-culture to reach mid-log phase). Then, five aliquots were added to 50 mL of fresh LB broth to an initial optical density measured at 600 nm (OD_600nm_) of 0.01. Each sample was treated with a specific CIP concentration of 0.0 (control), 5.0, 12.5, 25.0 or 50.0 µg/mL (final concentrations). Growth of cultures was monitored by OD_600nm_ at times 0, 2, 4, 6, 8, 12 and 16 h. Comparison of different CIP concentrations was done by assessing growth curve kinetics, including lag and exponential phases. As a complementary assay, evaluation of two other antibiotics was done in exactly the same growth conditions, but antibiotic concentrations depended on the MIC: imipenem (carbapenem) and tobramycin (aminoglycoside). See results and supplementary Figure S1 for details.

The growth curves were statistically compared to the control growth curve using a two-way ANOVA with Bonferroni post-tests (significance level of 95%), similar to^49^, using the time and concentrations as factors. We also ran a unpaired t-test (95% significance) comparing area under curve (AUC) of each growth curve against the control, similar to^50^. Analyses were done using Prism (GraphPad Software, Inc., La Jolla, CA). To perform the transcriptomic assay, we used the results from growth curves to select a specific sub-inhibitory CIP concentration at which there were no major changes in the growth rate after treatment.

### RNA isolation and RNA sequencing

In order to evaluate the molecular response of PaeAG1 to a sub-inhibitory CIP concentration, a transcriptomic assay was designed using RNA-Seq technology, as described below.

#### Growth conditions

PaeAG1 cells were grown under the same conditions as detailed before but treatment was done using a single CIP concentration of 12.5 µg/mL (see “Results” for details of concentration selection). Immediately after adding treatment, an aliquot was taken as control (time 0 h), and cells were kept growing for 2.5 and 5 h (times were selected according to preliminary results of phage induction, see “Methods” for Phage plaque assays). This was done with three independent cultures for a total of nine aliquots, three replicates per time.

#### RNA isolation

Aliquots from the cultures were preserved in two volumes of RNA protect reagent (QIAGEN) and cells were stored at 4 °C until RNA extraction. At the end of the sample collection period, total RNA was extracted using the RNeasy Mini kit (QIAGEN, UK) following the manufacturer’s instructions. RiboZero Gold (Epicentre) was used to deplete bacterial rRNA from total RNA samples according to manufacturer’s instructions. The quality and quantity of extracted RNA was determined using a Nanodrop (Nanodrop 2000, Thermo Scientific, UK). The RNA integrity was analyzed using Agilent 2,100 Bioanalyzer (Agilent Technologies, USA) to obtain the RNA integrity number (RIN) for all samples.

#### RNA sequencing

For RNA sequencing, TruSeq Stranded Total RNA library preparation kit (Illumina, USA) was used to generate cDNA (amplification with 13 PCR cycles) and libraries for 2 × 51 bp paired-end reads. Libraries were prepared and sequenced at the Genome Technology Center, New York University (New York, USA) on the Illumina HiSeq 2,500 platform. Sequencing generated more than 120 Gb of sequences (> 300 millions of reads in total) for all samples.

### RNA-Seq data analysis

With the aim of quantifying transcripts and identifying DEGs in PaeAG1 after CIP treatment, RNA-Seq data was analyzed including a quality control step, reads mapping to genome for transcript quantification and differential expression analysis.

#### Quality control (QC)

QC was done before and after trimming/filtering. Reads were trimmed using Trimmomatic v0.38^51^ to discard sequences with per base phred sequence quality score < 30 and 35 minimum length. Reads were filtered using BBDuk (https://jgi.doe.gov/data-and-tools/bb-tools/) to remove adapters and reads mapping to rRNA. Sequence files were evaluated using FastQC v0.11.7^52^ to obtain general quality control metrics. To evaluate the origin of reads sequences, FastQ-Screen^53^ was used to quantify the proportion of reads that mapped to reference genomes (human, mouse, and adapters contaminants, included by default) and prokaryotic sequences specifically added for this work (PaeAG1 and *E. coli* genomes, and rRNA 16S and 23S databases). Reports were merged using MultiQC^54^ to summarize all individual results. After selection, sequences for each of the nine samples had an average output of approximately 60 million reads.

#### Reads mapping and transcript quantification

We used EDGE-pro v1.3.1 software to: map RNA-Seq reads to the PaeAG1 genome (Genbank CP045739), filter out multialigned reads, and estimate expression levels of each gene by counts^55^. This program was run with the default parameters, using Bowtie2^56^ as read alignment algorithm. The script “edgeToDeseq.perl”, provided with the software, was used to convert raw counts (EDGE-pro output) to a count-table format for further differential expression analysis. Quality control of alignments per sample was done using: Qualimap RNA-Seq tool^57^ to assess mapping quality, and RSeQC package^58^ to estimate transcripts coverage uniformity (gene body coverage) and transcript integrity number (TIN). Required formats of genome annotation files for these analyses are available in https://github.com/josemolina6/PaeAG1_genome.

#### Differential expression analysis

We used raw counts of transcripts to estimate differential expression. For this purpose, DESeq2 package v1.26.0^59^ in R program v3.5.1^60^ was used based on the negative binomial generalized linear models, using default settings. DESeq2 based normalization, absolute expression comparisons by the regularized log transformation (rlog), Principal Component Analysis (PCA), counts dispersion plots and clustering analysis were run in the same program. Triplicates of each time after PaeAG1 exposure to CIP were considered as a factor level. Differential expression analysis was done comparing 2.5 h or 5 h data against the initial time point at 0 h. Hypothesis testing to select differentially expressed genes (DEGs) was done using Benjamini–Hochberg adjustment (to control false discovery rate, FDR) and log_2_[FoldChange] (logFC) of transformed and normalized mean counts. Genes were considered up-regulated if logFC > 1 or down-regulated if logFC < -1, considering an adjusted *p*-value < 0.05 for both cases. Gene list comparisons by Venn diagrams were performed using the Draw Venn Diagram Tool (https://bioinformatics.psb.ugent.be/webtools/Venn/).

### Annotation of differentially expressed genes

DEGs annotation was retrieved from our previous work^5^ for the assembly and annotation of PaeAG1 genome (Genbank CP045739). Particular features per gene (including molecular function, product, gene size and domains, and sub cellular location of proteins) were explored in more detail from *Pseudomonas* Genome Database (https://www.pseudomonas.com/)^61^. In addition, general regulators of the DEGs were investigated using PseudomonasNet tool (https://www.inetbio.org/pseudomonasnet/Network_regulon_form.php) with a *p*-value < 0.05 in a context-centric analysis. Using the same platform, it was possible to identify the DEGs and their regulators that corresponded to transcription factors genes.

### Analysis of DNA–protein interactions

For selected genes, protein-DNA binding sites were investigated. The CollectTF database (https://www.collectf.org/) was primarily used to search for consensus DNA binding sequences of the protein of interest and to identify modulated genes. If no information was available, promoter consensus sequences were searched from particular studies and the identification of binding sites was done using the motif-based sequence analysis tool (MEME, using Find Individual Motif Occurrences FIMO, https://meme-suite.org/tools/fimo).

In order to identify DEGs as molecular determinants (hub genes, gene clusters and key pathways) of the response to CIP in PaeAG1, a large scale gene–gene interaction (GGI) network of DEGs was built using a top-down systems biology approach. Connections between genes were predicted using two independent methods, one using a database-based model and another from co-expression analysis, detailed as follows.

### Database-based method for gene–gene interactions prediction and network construction

With the aim of obtaining a high confidence GGI between DEGs using a database-based method, the Search Tool for the Retrieval of Interacting Genes database (STRINGdb)^62^ was used to construct a large scale GGI network for the DEGs using default parameters. All DEGs at any of the two times were used to build the main network. The resulting graph was exported and then visualized and topologically analyzed using Cytoscape software^63^.

### Co-expression analysis and co-expression network construction

To incorporate more interactions between DEGs, a data-driven systems biology approach was implemented using co-expression analysis with all the normalized counts of DEGs, as in recent studies^45,64–66^.

#### Modules identification using co-expression analysis

Weighted gene co-expression network analysis (WGCNA) package^43^ was run in R software. Briefly, a matrix of Pearson correlation between all pairs of genes was calculated. The adjacency matrix was then constructed using a power of *β* = 9 as a saturation level for a soft threshold of the correlation matrix based on the criterion of scale-free topology. The topological overlap matrix was calculated. Hierarchical clustering was used to generate a dendrogram to group highly co-expressed genes, creating gene clusters called modules (arbitrarily represented by colors) using the default dynamic tree cut algorithm. Default colors given to modules were kept.

#### Association of co-expression modules and traits

A t-test evaluated the association between the modules (using module eigengene ME, the first principal component gene of module expression matrix) and traits of PaeAG1 according to the experimental design. For this, the times (the experiment factors 0, 2.5 and 5 h) and data of phage induction at 2.5 and 5 h after 12.5 µg/mL CIP exposure were incorporated as traits (see “Phage plaque assay” section in “Methods”).

#### Co-expression network

To visualize the whole network including the modules by colors, the WGCNA “exportNetworkToCytoscape” function was run, using a correlation threshold of 0.985 and weight = false to build an un-weighted graph of highly connected genes with very strict correlation. The data-driven graph was visualized using Cytoscape.

### Integrated DEGs network construction

The final GGI network of DEGs was constructed joining the files of the well-known interactions predicted by STRING database and the strict data-driven interactions obtained from co-expression analysis (un-weighted graph). The definitive graph was visualized using Cytoscape software. Topological metrics of the graph were obtained using the defaults apps available in Cytoscape.

### Enrichment analysis

For the gene set enrichment analysis (GSEA), STRINGdb was used to identify significantly enriched pathways according to KEGG database, using a cutoff of FDR < 0.05. This analysis was run for complete gene lists of DEGs at 2.5 h, DEGs at 5 h, and genes of each co-expression module. Results of enrichment were incorporated into the DEGs network using the Cytoscape app Omics Visualizer (https://apps.cytoscape.org/apps/omicsvisualizer).

### Hub genes identification

In order to identify central or hub genes in the DEGs network of PaeAG1 after exposure to CIP, cytoHubba app^67^ was run in Cytoscape. To address this, bottleneck and betweenness methods were implemented with default parameters. The top 10 nodes (genes) were selected for each method using calculated metrics. All selected genes in any of the methods were labeled as hub genes. In addition, cytoHubba was also used to build two subnetworks using the hub genes, one with the selected elements only, and another including the first-stage nodes (in direct connection with hub genes) to identify gene clusters. KEGG annotation information was kept from the DEGs network.

Expression profiles of hub genes were compared to expression levels obtained in other representative studies, including the following stressors: Cu (copper)^68^, CIP (ciprofloxacin)^26^, COL (colistin)^69^, AZM (Azithromycin)^70^ and H_2_O_2_ (hydrogen peroxide)^71^. Comparison was done using the general information of expression levels (down, up or variable regulation).

### Phage plaques assay (validation assay at the phenomic level)

To validate the transcriptomic results which showed an up-regulation of phage genes in PaeAG1 after exposure to CIP, we implemented a phage plaques assay and performed this assay in triplicate. To assess the CIP effect on phage induction, different CIP concentrations were evaluated. Evaluation was also done for imipenem and tobramycin as supplementary assays. Growth conditions were the same as described in the “Growth curve assays”, until the addition of different antibiotic concentrations. At this point, cultures were kept growing for five hours and phages were isolated and quantified for each sample. During standardization, it was determined that five hours after CIP exposure was the minimum time for clear detection of phage plaques (see supplementary Figure S1-B for details). Phage plaque counts at 2.5 h and 5 h for 12.5 µg/mL CIP were used to associate the phage induction with co-expression modules (detailed in “Co-expression analysis” section).

#### Phages isolation

Protocols of ^72^ and ^73^ were adapted. Briefly, the culture was centrifuged for 20 min at 4,000 rpm, 40 mL of the supernatant was taken and 1 mL of chloroform was added to residual bacterial cells. After overnight incubation, cell debris was removed by centrifugation for 20 min at 3,000 rpm. The supernatant was filtered through a 0.45 μm filter to select phages. A volume of 30 mL of the filtered supernatant was mixed with 7.5 mL of polyethylene glycol (20%) and NaCl (2.5 M) to precipitate the phages. After overnight incubation, the sample was centrifuged for 30 min at 4,000 rpm, the supernatant was discarded and the pellet was resuspended in 250 µL of phage buffer (10 mM MgSO_4_, 10 mM Tris–HCl and 150 mM NaCl).

#### Phages quantification

Phages were quantified by means of Plaque Forming Units (PFU) using *P. aeruginosa* PAO1 as host cells. The numbers of PFU was determined using the double-agar-layer method^74^. Briefly, medium was composed of two agar layers, a first layer 1.5% and another to 0.5% agar concentration. *P. aeruginosa* PAO1 and phages were added on the second layer and phage plaques were visualized after incubation for 24 h at 25 °C.

An exponential regression between the CIP concentrations and the PFU was run to associate the effect of CIP exposure on the phage induction.

### Ethical considerations

No animals or human participants were included in this study. Both the scientific committee of the Centro de Investigación en Enfermedades Tropicales (CIET) and Vicerrectoría de Investigación of Universidad de Costa Rica approved the study and the access to the PaeAG1 strain from the CIET collection of bacterial specimens.

## Results

### Concentration-dependent effect of CIP compromises the growth rate of PaeAG1

**Figure 2:**
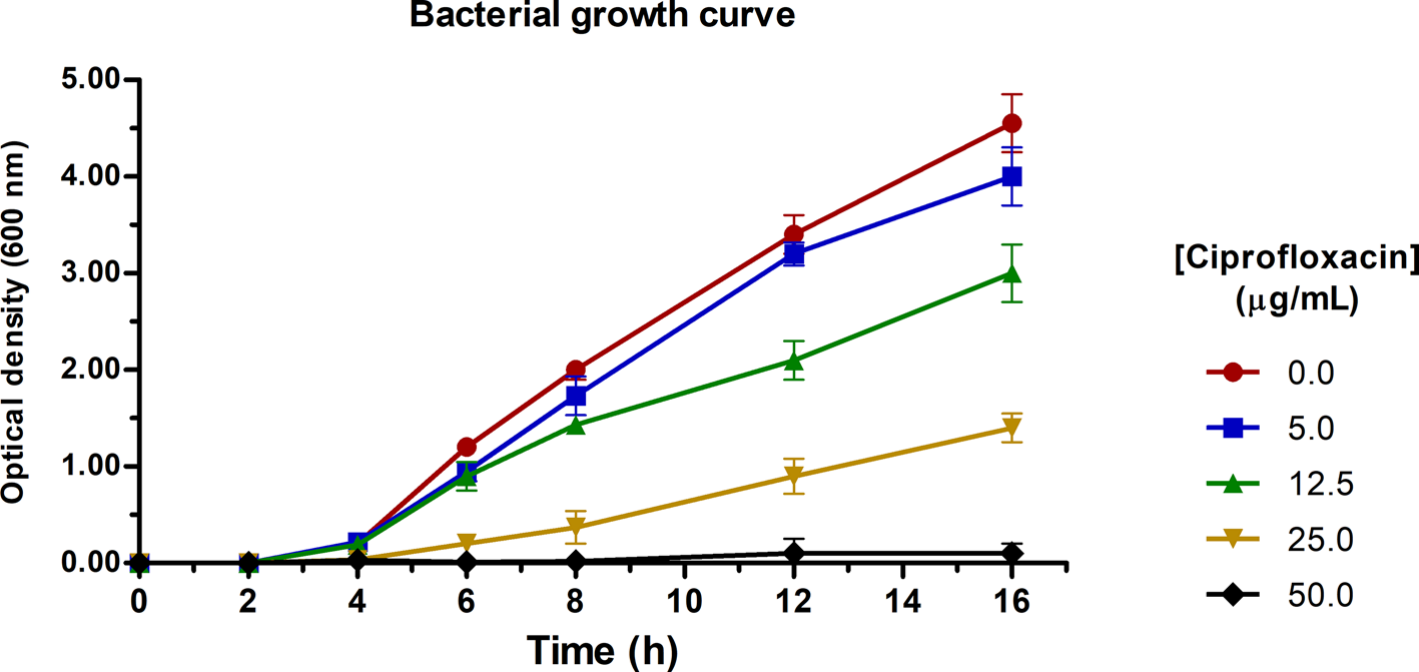
In vitro effects of ciprofloxacin on growth curve of PaeAG1. A growth rate reduction was observed as the CIP concentration was incremented. Area under curve (AUC) was compared using t-test (p < 0.05), showing a statistical difference between all curves when compared to control (0.0 mg/mL). In a similar manner, two-way ANOVA found differences in the OD_600nm_ and time for each case.

To evaluate the effects of CIP in the growth rate of PaeAG1, increasing concentrations of the antibiotic were added to exponential-phase PaeAG1, and growth was monitored over time for 16 h. As shown in Fig. 2, OD_600nm_ values were highly consistent between replicates (error bars represent standard deviation). All CIP curves showed a statistical significant difference on OD_600nm_ compared to control (p < 0.05 for both AUC and two-way ANOVA). Lag phase for the control and two lower CIP concentrations (5 and 12.5 µg/mL) lasted approximately 4 h, while the higher CIP concentration of 25.0 µg/mL showed a lag phase of 8 h.

Kinetics at the exponential phase showed more variable results. There was a decrease in cell growth for 12.5 µg/mL CIP from 12 h onwards in comparison to 0 or 5.0 µg/mL, and more evident at same time for 25 µg/mL. For the case of 50.0 µg/mL (higher than MIC), the growth was drastically impaired and no exponential growth was observed. These results indicate that higher CIP concentrations have a stronger effect on the growth rate, even for sub-inhibitory concentrations (MIC_Ciprofloxacin_ 32 µg/mL). Evaluation of the growth effects of other two antibiotics (imipenem and tobramycin) was also performed (supplementary Figure S1C–E, left). Unlike CIP, both cases showed no changes in the growth curves with different sub-inhibitory concentrations.

Due to the significant changes in growth curves with CIP (with respect to control) and considering a condition with enough cell mass for RNA-Seq analysis, 12.5 µg/mL CIP was used to evaluate the transcriptomic response of PaeAG1 to a sub-inhibitory concentration of the antibiotic.

### RNA-Seq analysis identifies 518 DEGs in PaeAG1 over time after exposure to CIP

A transcriptomic analysis was conducted to evaluate the molecular response to sub-inhibitory CIP concentration in PaeAG1. To this end, samples were taken at 0 (control), 2.5 and 5 h after CIP treatment. To ensure exponential growth at these times, the growth curve was monitored using OD_600nm_ measurements (successfully reproduced as Fig. 2), in addition to counting of Colony Forming Units (CFU), as shown in supplementary Figure S1A. After RNA was extracted, RNA integrity RIN > 9 was obtained for all samples and paired-end RNA sequencing was performed. For all samples, quality control of raw sequence data showed good results in terms of mean quality (> 30), no adapters, and no reads mapping to rRNA after filtering. Read mapping quality control showed that 98.6% were mapped to the PaeAG1 genome, with expected uniform coverage for gene body, and TIN > 90 for all samples. Details of assessment of transcriptomic data (counts per gene) is shown in supplementary Figure S2.

**Figure 3:**
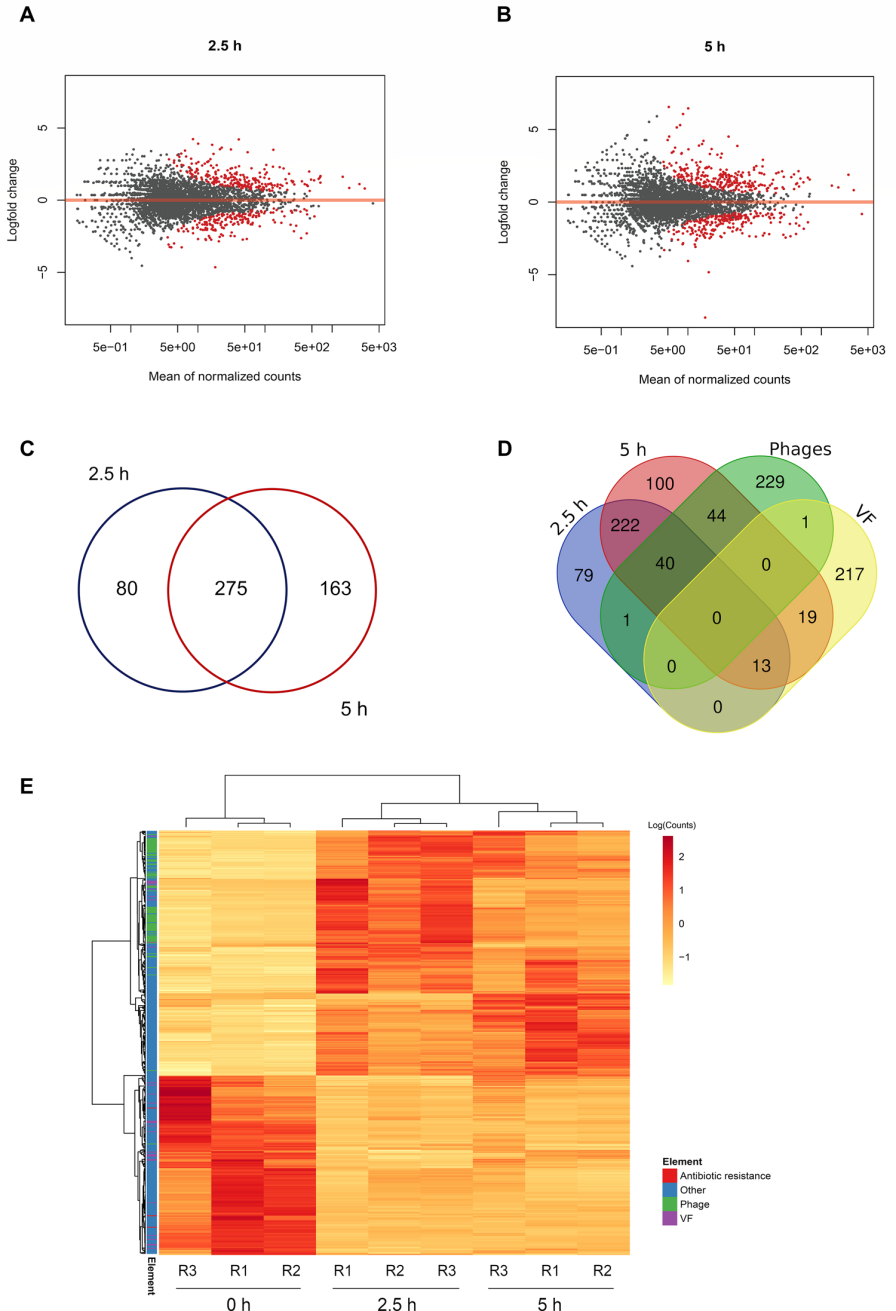
Differential expression analysis in PaeAG1 exposed to ciprofloxacin compared to initial time 0 h. Selection of DEGs according to adjusted *p*-value (p < 0.05) and logFC (logFC < − 1 or logFC > 1) at 2.5 h (**A**) or 5 h (**B**) post-exposure to antibiotic. (**C**) Venn diagram showing the comparison of DEGs in the two evaluated times, with 275 shared genes (intersection) and total 518 genes at any time (union) with respect to time 0 h (control). More details in Table 1. (**D**) Venn diagram showing the comparison of DEGs and phage genes or virulence factors (more details in Table 2). (**E**) Heatmap of normalized counts and gene clustering of the total 518 DEGs at the three evaluated time points.

Identification of DEGs was conducted by comparing times 2.5 or 5 h against the initial 0 h time after CIP exposure (Fig. 3A,B). As shown in Table 1, 355 DEGs were identified at time 2.5 h, with 204 (57.5%) up-regulated and 151 (42.5%) down-regulated. At 5 h, 248 (56.6%) genes were up-regulated, meanwhile 190 (43.4%) were found to be down-regulated, for a total of 438 DEGs.

**Table 1:** Comparison of DEGs of PaeAG1 at 2.5 and 5 h after treatment with Ciprofloxacin, including counts of down or up regulated genes, shared genes (intersection) and total genes at both times (union).

DEGs	Sets			
	2.5 h	5 h	2.5 h ∩ 5 h	2.5 h ∪ 5 h
Up regulated genes	204	248	153	299
Down regulated genes	151	190	118	223
Total DEGs	355	438	275	**518**

**Figure 4:**
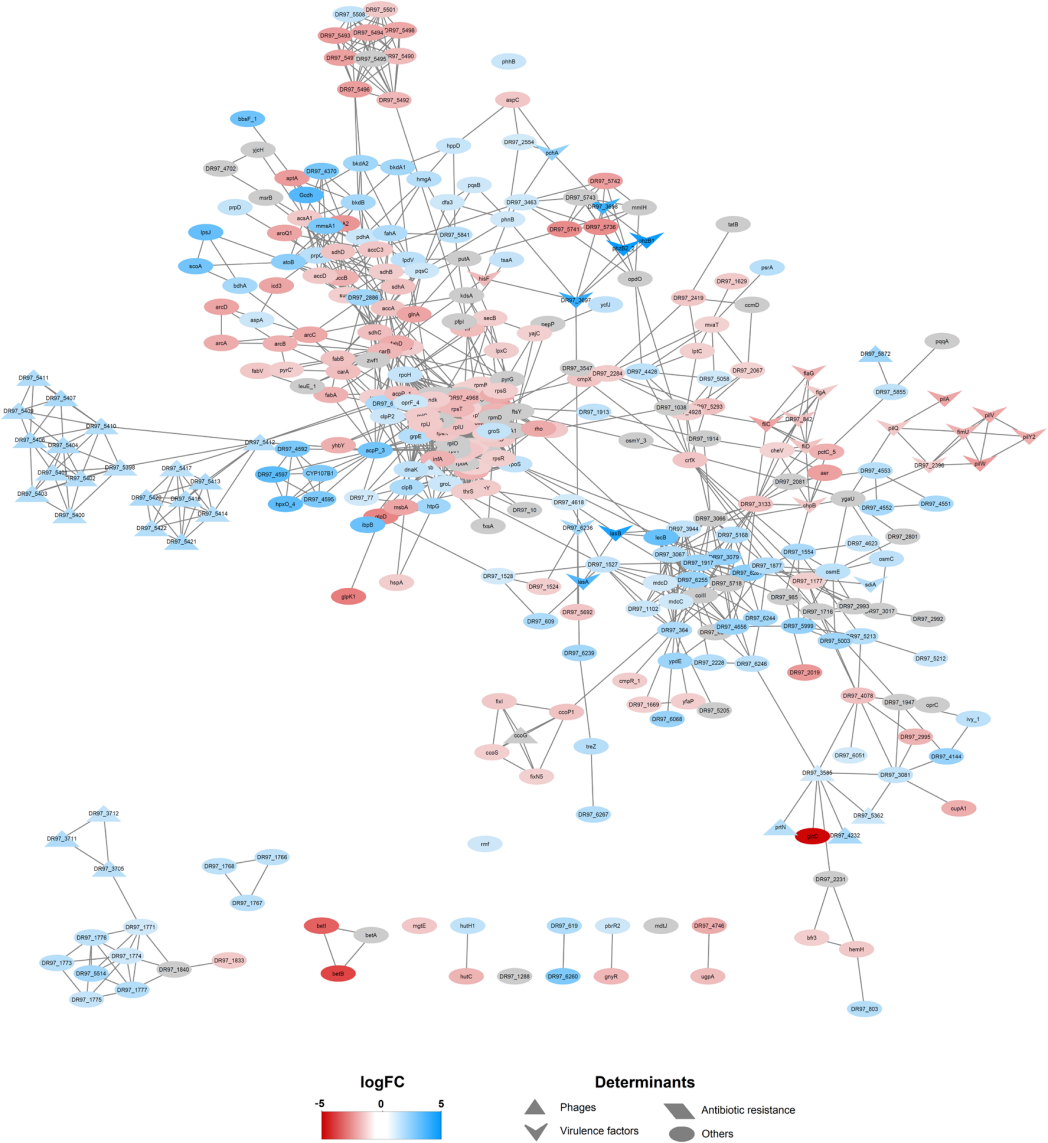
Gene–gene interaction (GGI) large scale network of differentially expressed genes in PaeAG1 after ciprofloxacin treatment, using a database-based method for prediction of interactions. Using STRINGdb, interactions between genes were predicted. To build the network all the DEGs in both times 2.5 and 5 h were included. A total of 342 genes resulted connected (66.0% of all DEGs) with 1685 edges in total (not connected nodes are not shown). The logFC is shown for 5 h. Gray nodes represent genes that were differentially expressed only at time 2.5 h (i.e. no logFC value is displayed at time 5 h). Details of the network by time is shown in supplementary Figure S3. Phages genes, virulence factors and antibiotic resistance genes are represented as triangles, arrowheads and rhomboids, respectively. Down-regulation (red tones) and up-regulation (blue tones).

A total of 518 DEGs were found at any time points (union ∪), as shown in Fig. 3C and Table 1. These represent around 7% of the genes of PaeAG1. In addition, as presented in Fig. 3D, a total of 85 DEGs (at any time) belong to phages (27.6% of the 308 phage genes identified in the PaeAG1 genome), most of them up-regulated as shown in Table 2, Fig. 4 and supplementary Figure S3. The phages regulated include phiCTX, F10, JBD44 and JDO24 for which 3, 10, 65 and 7 DEGs were respectively observed at any time (Table 2).

**Table 2:** Comparison of DEGs of PaeAG1 at 2.5 and 5 h after treatment with ciprofloxacin, and specific phages or categories of virulence factors, including shared genes (intersection) and total genes at both times (union), the regulation and the type of elements.

Determinants			Sets of DEGs				
Type	Specific elements	Total genes (in PaeAG1 genome)	2.5 h	5 h	2.5 h ∩ 5 h	2.5 h ∪ 5 h	Regulation* and observations
Antibiotic resistance	Total	56	3	2	2	3	Down, lactamases
Phages	PPpW	12	0	0	0	0	No DEGs
	phiCTX	25	2	3	2	3	Up
	F10	62	1	9	0	10	Up
	JBD44	105	34	65	34	65	Up
	JDO24	59	4	7	4	7	Up
	phi3	45	0	0	0	0	No DEGs
	Total	308	41	84	40	85	–
Virulence factors	Adherence	96	11	19	11	19	Down
	Antimicrobial activity	17	1	6	1	6	Up, phenazines
	Antiphagocytosis	25	0	0	0	0	No DEGs
	Phospholipases	3	0	0	0	0	No DEGs
	Biosurfactant	3	0	0	0	0	No DEGs
	Iron uptake	28	0	1	0	1	Up, Pyochelin
	Protease	4	1	2	1	2	Up, elastases
	Quorum sensing	5	0	1	0	1	Up, RhlR
	Regulation GacS/GacA system	2	0	0	0	0	No DEGs
	Secretion system	63	0	2	0	2	Down, T3SS
	Toxins	4	0	1	0	1	Up, hydrogen cyanide
	Total	250	13	32	13	32	–

*Based in logFC of genes for both times 2.5 and 5 h. Type of elements is also shown.

In the case of the 250 known virulence factors of PaeAG1, 32 (12.8%) were identified as DEGs at all of the assessed times (arrowheads of Fig. 4 and supplementary Figure S3). The virulence factors are mainly associated with adherence (19) and phenazines (6) genes (see Table 2). Regarding antibiotic resistance genes, only three out the 56 genes were found to be differentially expressed (Table 2).

A heatmap of normalized counts and gene clustering of the total 518 DEGs are shown in Fig. 3E. Well-defined clusters were found for genes and samples, showing similar expression patterns.

Out of all the DEGs at 2.5 h, seven genes corresponded to transcription factors, including *psrA, rpoH* and *prtN*. At 5 h, 14 DEGs including *psrA*, *rpoH*, *prtN*, *rpoS*, *rhlR* and *ptrB* were identified as transcription factors. All transcription factors activated at 2.5 h remained active at 5 h (Supplementary Table S2). Identification of regulators by a context-centric analysis revealed a total of 22 transcription factors modulating all the DEGs at 2.5 h, and most of them are part of the 28 transcription factors recognized as DEGs at 5 h (see Supplementary Table S2).

Genes of the SOS response were not identified as DEGs. The *rpoS* factor was up-regulated at 5 h. Due to the preponderant role of LexA (SOS response) and RpoS as essential genes in the response to CIP in *P. aeruginosa*, we further investigated the DNA binding sites for these elements. The CollectTF database provided the consensus binding sequence for LexA as CTG-TATAA-ATATA-CAG, described by^26^. Analysis revealed the role of LexA modulating all 15 genes in the SOS response in *P. aeruginosa,* as well as other sequences at promoter regions of *psrA* (coding for a transcription factor as described before)*, grpE, hemO* and other genes. In PaeAG1, *psrA* and *grpE* genes were up-regulated at 2.5 and 5 h after CIP treatment. For RpoS, no sequence information was available in CollectTF, therefore we used the RpoS-dependent promoter consensus sequence CTATACT found by^75^. A total of 49 sites for RpoS were predicted to be associated with promoter regions of PaeAG1 genes, but none as DEGs in PaeAG1.

### Networks analysis shows pleiotropic effects of CIP exposure in PaeAG1

Using a top-down systems biology approach, a large scale GGI network of DEGs was built to identify molecular determinants associated with the response to CIP in PaeAG1.

*GGI predictions by a database-based model:* All of the 518 DEGs were incorporated as nodes and edges (high confidence connections or interactions). A total of 342 (66.0% of all DEGs) nodes were found to be connected with at least one other gene, as well as 1685 edges were established (Fig. 4). When selecting DEGs for each time, 248 nodes (69.9%) of the 355 DEGs at 2.5 h were connected with a total of 1,156 edges (supplementary Figure S2A). Out of all the 438 DEGs at 5 h, 284 (64.8%) were connected with 1,041 edges in total (supplementary Figure S2B).

As shown in Fig. 4, some determinants of virulence factors (adherence) and antibiotic resistance genes showed a down-regulation after CIP treatment, meanwhile, phage genes and other virulence factors (phenazines) were found to be up-regulated. In addition, gene clusters of highly connected DEGs showed the same expression pattern, suggesting a coordinated regulation.

The observed unconnected genes (107 DEGs for 2.5 h and 154 for 5 h) are inherent to limitations in the database (incomplete inclusion of phage genes) or the current state of the gene annotation (without information, hypothetical protein, etc.). To improve the associations between genes creating more connections, a data-driven co-expression analysis was run.

#### Co-expression analysis

**Figure 5:**
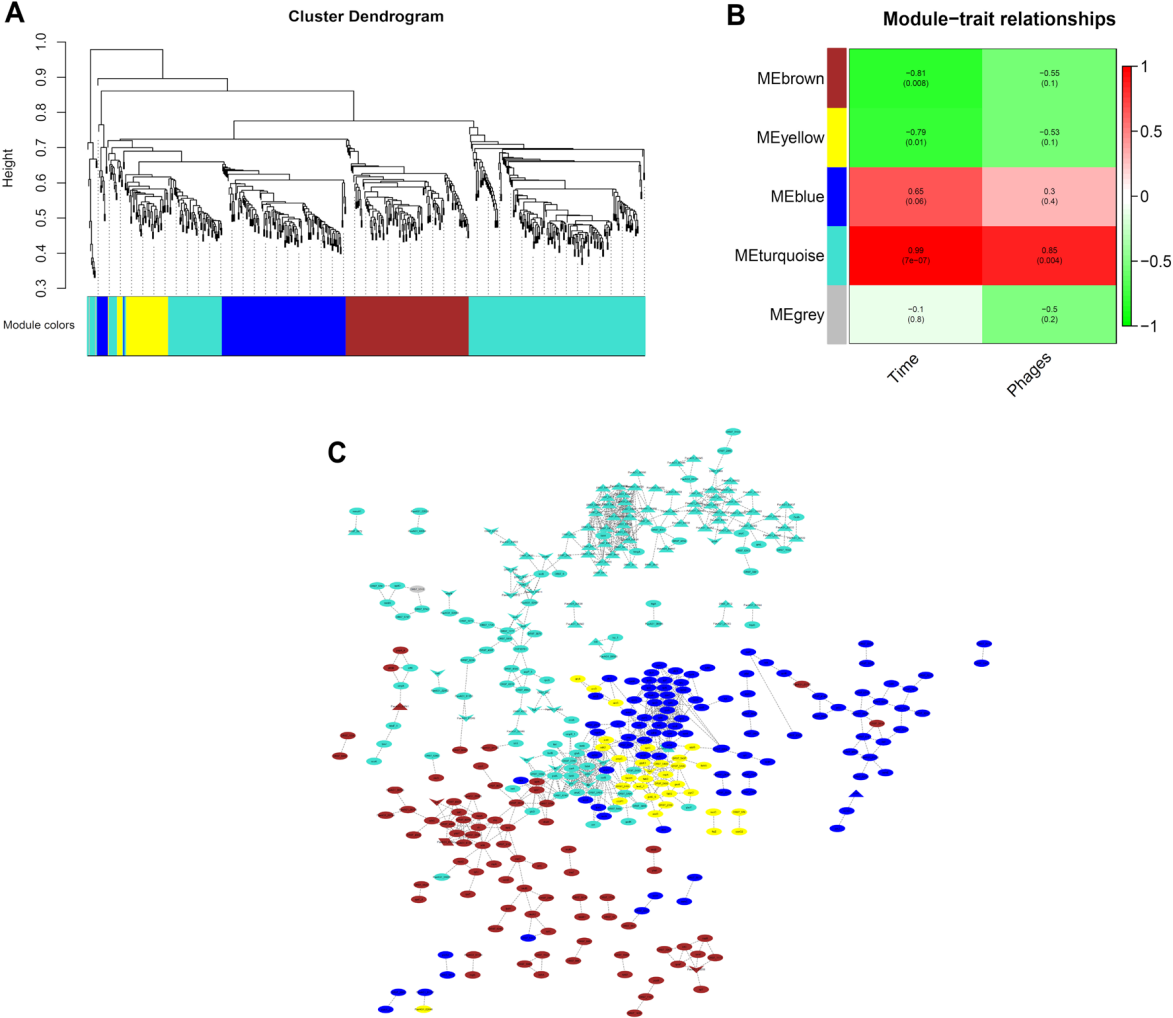
Co-expression analysis to identify modules of genes and the data-driven co-expression network in PaeAG1 after Ciprofloxacin treatment. (**A**) Modules identification (clusters by colors) using correlated expression genes (along times 0, 2.5 and 5 h) and clustering analysis after WGCNA was implemented. (**B**) Association of modules to traits, showing relations between turquoise and blue modules with exposure time to antibiotic and phages induction. (**C**) Data-driven co-expression network using correlation of gene expression by WGCNA analysis (correlation > 98.5%). A total of 388 DEGs were found to be connected, with a total of 1,073 edges. Only correlated genes are shown. More details in supplementary Figure S3A. Phages genes, virulence factors and antibiotic resistance genes are represented as triangles, arrowheads and rhomboids, respectively.

Modules of highly connected genes (represented using color groups) were created using normalized counts for all the 518 DEGs. As shown in Fig. 5A, genes were clustered into four main modules, showing similar expression along samples. The number of genes belonging to the turquoise module was 239, 124 for blue, 114 brown and 39 for yellow module. In the co-expression network (Fig. 5C), a total of 388 DEGs (74.9% of the 518 DEGs) were found to be connected, with a total of 1,073 edges. Of these interactions, 385 were also found using the database-based model and 688 novel gene interactions were suggested by our co-expression analysis. The turquoise module includes most of the phage genes and virulence factors.

#### Integrated GGI network of DEGs

**Figure 6:**
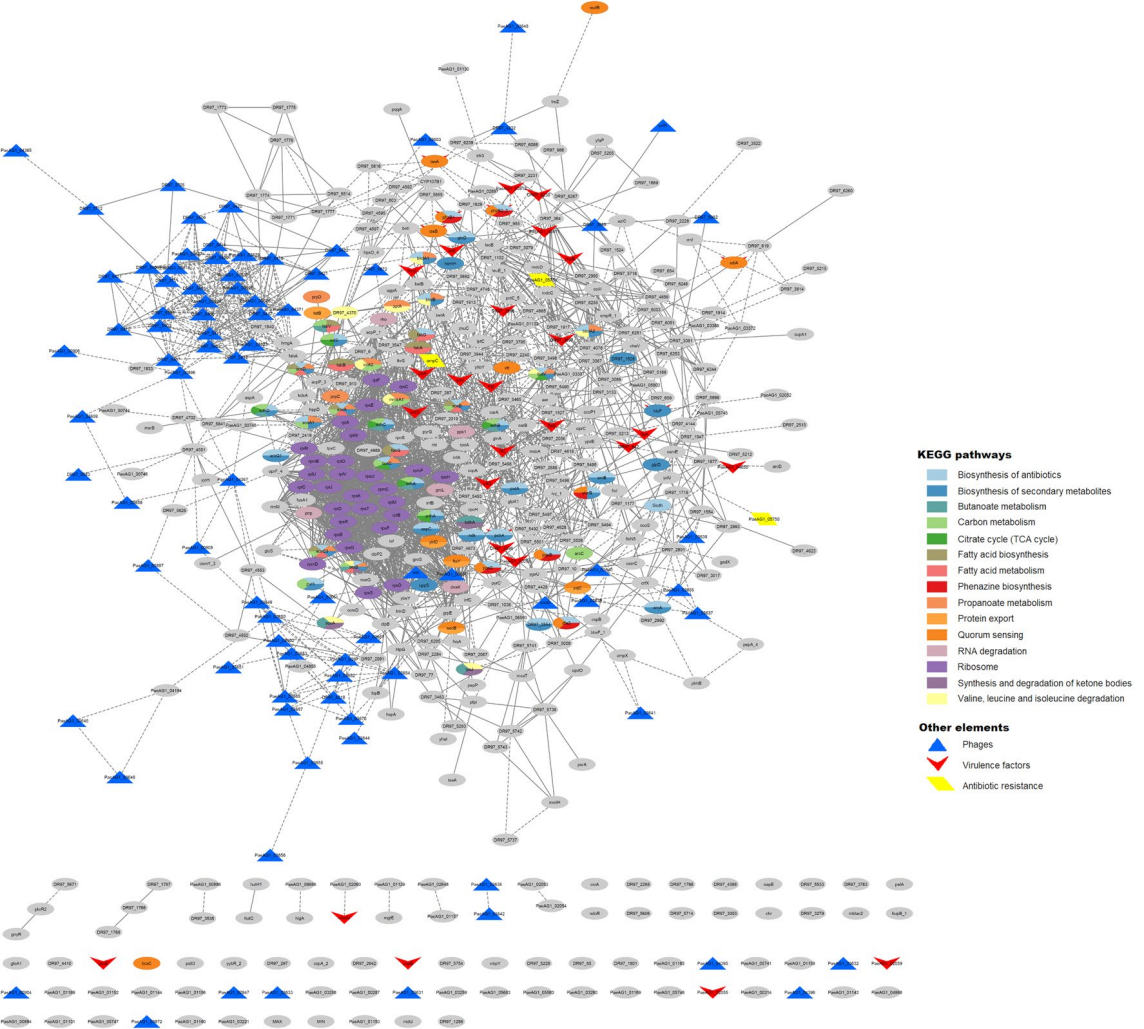
Definitive large scale network of DEGs, identification of hub genes and associated groups in PaeAG1 after treatment with ciprofloxacin. Network showing all 518 DEGs genes and their interactions (449 genes have at least one connection). Known interactions according to STRINGdb (database-based method) are shown as solid lines and data-driven interactions according to data-driven co-expression analysis as dashed lines. Enriched nodes associated to KEGG annotation are colored according to each pathway (more details in Table 3). Phages genes, virulence factors and antibiotic resistance genes are represented as triangles, arrowheads and rhomboids, respectively. Other genes are represented as ellipses.

Integration of predicted connections between genes by both the database-based model and co-expression analysis was done to build a definitive large scale network, shown in Fig. 6. A total of 449 (86.7%) of DEGs were connected, in contrast with the 342 nodes from the preliminary network, an increment of ~ 20%. In addition, 2,373 edges were identified, 1685 from the database-based method (solid lines in the network) and the 688 new interactions suggested by the co-expression analysis (dashed lines). Furthermore, a separated cluster was observed with high connectivity between phage genes (cluster of blue triangles, Fig. 6 left top). Remarkably, this cluster appears to have a critical bottleneck at the *fahA* gene, since many genes are connected to this node but, for the majority of the cluster nodes, this gene is the only connection to the rest of the network. Thus, the cluster becomes a clearly separated module. In addition, another smaller and less distinct cluster of phage genes was formed (Fig. 6 left down).

The same GGI network is presented in supplementary Figure S4A to show the distribution of genes by co-expression modules. A high functional interaction of genes across different clusters is observed. The logFC values at time 5 h are shown in the network in Figure S4B.

#### Enrichment analysis

**Figure 7:**
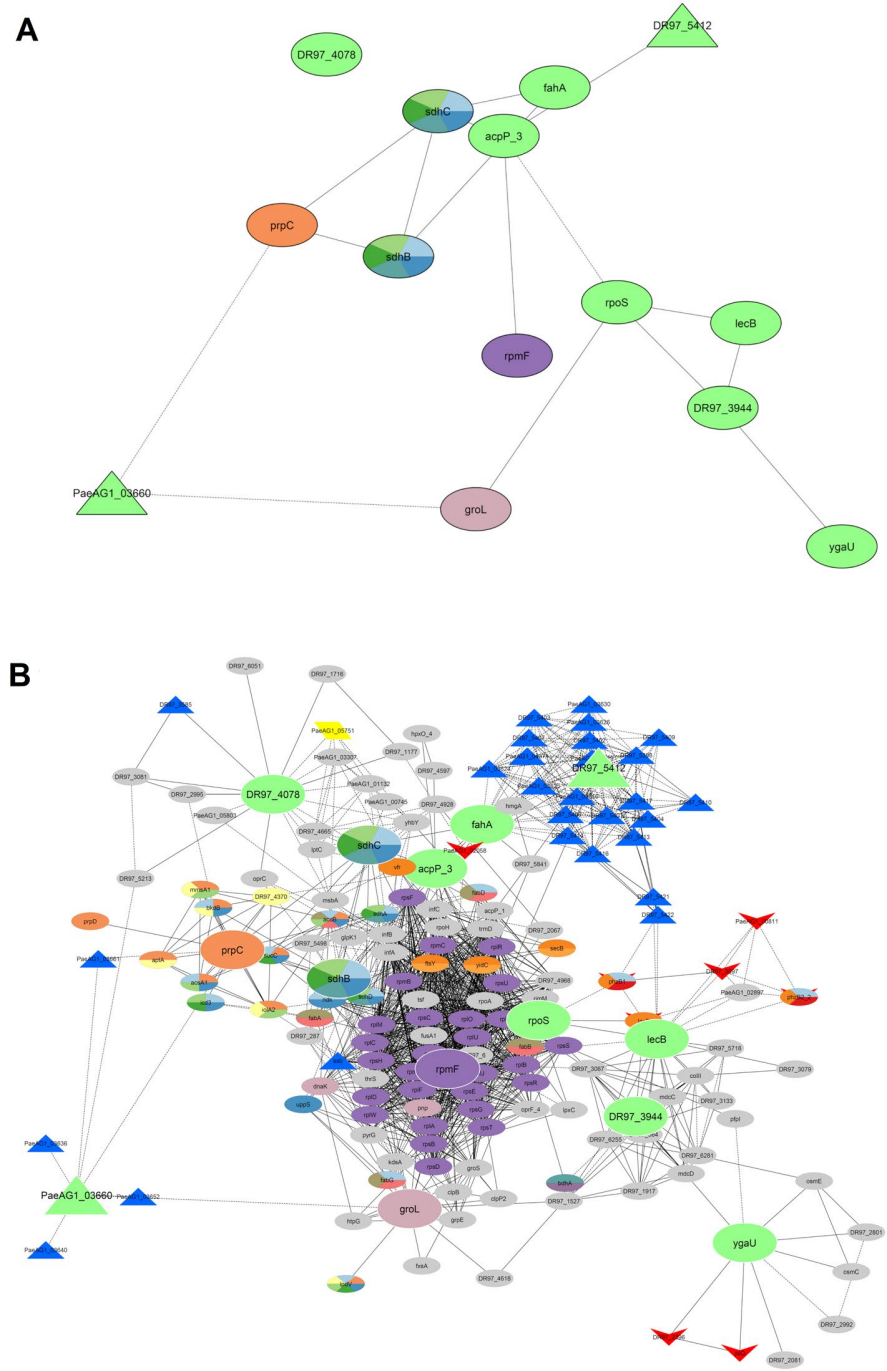
Identification of hub genes and first-stage subnetwork of their associated groups in PaeAG1 after treatment with ciprofloxacin. (**A**) Hub genes identification using cytoHubba (betweenness and bottleneck methods) in the network of DEGs (large nodes). Details in Table 4. (**B**) Subnetwork of nodes that directly interact with the 14 hub genes were used to build a first-stage elements network. Details of node shapes and colors are the same as described in Fig. 6.

In order to gain insight about the biological meaning of DEGs, gene set enrichment analysis (GSEA) was performed. The 518 DEGs were shown to be implemented in a total of 15 KEGG pathways (Figs. 6 and 7, and Table 3). The enriched pathways included ribosomal functions, RNA degradation, biosynthesis of antibiotics, fatty acids metabolism, propanoate metabolism, fatty acids biosynthesis, quorum sensing, amino acid degradation, carbon metabolism and citrate cycle, butanoate metabolism, phenazine biosynthesis, among others (see Fig. 7). Details of gene counts, FDR and regulation are shown in Table 3. Additionally, pathways by co-expression modules (Table 3) showed that some of them are enriched in specific pathways. For example, the blue module is down-regulated for ribosomal activity and RNA degradation (exclusive functions for this module), meanwhile the yellow module has multiple but tightly related pathways, most of them associated to interconnected metabolism pathways, down-regulated.

**Table 3:** Pathways related to DEGs network of PaeAG1 exposed to ciprofloxacin, according to KEGG annotation.

KEGG term ID	Term description	Total gene count	DEGs 2.5 h		DEGs 5 h		Modules	Regulation (% DEGs)*
			Observed gene count	FDR	Observed gene count	FDR		
paeb01130	Biosynthesis of antibiotics	266	30	0.0015	34	0.00047	Brown, Yellow	Down (61%)
paeb01110	Biosynthesis of secondary metabolites	320	30	0.0352	31	0.0205	Yellow	Down (70%)
paeb00650	Butanoate metabolism	37	8	0.0133	9	0.0068	Yellow	Down (55%)
paeb01200	Carbon metabolism	126	15	0.0258	18	0.0068	Yellow	Down (80%)
paeb00020	Citrate cycle (TCA cycle)	30	7	0.0158	8	0.0068	Yellow	Down (75%)
paeb00061	Fatty acid biosynthesis	27	7	0.0131	9	0.0014	Yellow	Down (100%)
paeb01212	Fatty acid metabolism	49	8	0.0309	10	0.0068	Yellow	Down (90%)
paeb00405	Phenazine biosynthesis	20	5	0.0309	6	0.0127	Brown	Up (100%)
paeb00640	Propanoate metabolism	47	12	0.00061	16	3.87e-06	Brown, Yellow	Variable (50/50)
paeb03060	Protein export	15	5	0.026	3	0.0014	Yellow	Down (100%)
paeb02024	Quorum sensing	86	11	0.0317	14	0.0068	Brown	Up (69%)
paeb03010	Ribosome	55	27	1.95e-14	27	2.63e-13	Blue	Down (100%)
paeb03018	RNA degradation	17	5	0.0258	5	0.0273	Blue	Down (60%)
paeb00072	Synthesis and degradation of ketone bodies	10	4	0.0258	4	0.0273	Brown, Turquoise	Up (100%)
paeb00280	Valine, leucine and isoleucine degradation	46	11	0.0015	11	0.0023	Brown, Turquoise	Up (82%)

Annotation of modules of co-expressed genes and the general regulation are also included.

*Based on logFC of DEGs at both times 2.5 and 5 h.

### Only 14 hub genes are able to represent the key pathways regulated by CIP in PaeAG1

With the aim of identifying an inter-modular key or central genes in the DEGs network of PaeAG1 after exposure to CIP, an analysis of hub gene identification was conducted. This approach revealed 14 connected hub genes (Fig. 7A and details in Table 4). Two genes, identified as PaeAG1_03660 and PaeAG1_03610, are part of the phage JBD44 and they were up regulated at 5 h. Topologically, they are part of the two identified phage gene clusters in the main network (Fig. 6). Two genes, *sdhB* and *sdhC,* (down-regulated) have functions related to carbon and butanoate metabolism, and biosynthesis of secondary metabolites. Interestingly, the ribosomal protein L32 (*rpmF*, down-regulated), a chaperonin (*groL*, up-regulated) and the sigma factor (*rpoS*, up-regulated) were also identified as single molecular determinants of the network. Also, the *fahA* gene, which was previously recognized as a bottleneck for the phage genes cluster and coding for fumarylacetoacetase enzyme, was identified as a hub gene.

**Table 4:** Characterization of hub genes in the DEGs network of PaeAG1 after treatment with ciplofloxacin.

PaeAG1 Locus ID	Gene name	Betweenness score*	Bottleneck score*	logFC 2.5 h*	logFC 5 h*	Co-expression module	KEGG Annotation**	Annotation details	Other studies***
PaeAG1_01864	acpP (PA2966)	6,268.3	17	2.64	3.63	Turquoise	Metabolic pathways, biosynthesis of antibiotics	Acyl carrier protein; fatty acid biosynthesis	↑ AZM, ↕ CIP COL
PaeAG1_06246	ygaU	6,340.4	–	1.79	0.9	Blue	–	LysM domain/BON superfamily protein	–
PaeAG1_04068	sdhB (PA1584)	6,401.4	–	− 1.37	− 1.17	Blue	Biosynthesis of antibiotics, Carbon metabolism, Citrate cycle (TCA cycle), Butanoate metabolism, Biosynthesis of secondary metabolites	Succinate dehydrogenase and fumarate reductase iron-sulfur family protein	↑ COL AZM ↓ CIP Cu
PaeAG1_04991	prpC (PA0795)	6,485.7	14	1.58	1.7	Turquoise	Propanoate metabolism	Belongs to the citrate synthase family	↑ H_2_O_2_ CIP ↓ AZM ↕ COL
PaeAG1_03610	DR97_5412	7,285.4	15	0.9	1.84	Turquoise	–	Phage: JBD44; Tail tape measure protein	–
PaeAG1_05221	groL or groEL (PA4385)	8,440.2	–	1.16	1.21	Turquoise	RNA degradation	60 kDa chaperonin; Prevents misfolding and promotes the refolding and proper assembly of unfolded polypeptides generated under stress conditions	↑ CIP Cu ↓ AZM ↕ H_2_O_2_
PaeAG1_04071	sdhC (PA1581)	8,716.8	–	− 1.68	− 1.54	Brown	Biosynthesis of antibiotics, Carbon metabolism, Citrate cycle (TCA cycle), Butanoate metabolism, Biosynthesis of secondary metabolites	Succinate dehydrogenase, cytochrome b556 subunit	↑ AZM ↓CIP Cu
PaeAG1_03660	PaeAG1_03660	9,477.2	17	1.05	1.23	Turquoise	–	Phage: JBD44	–
PaeAG1_03555	fahA (PA2008)	11,245.9	16	1.19	1.93	Turquoise	Tyrosine metabolism	Fumarylacetoacetase	↑ CIP ↕ COL ↓ Cu AZM
PaeAG1_01837	lecB (PA3361)	13,150.8	17	1.71	3.88	Turquoise	Quorum sensing	fucose-binding lectin PA-IIL	↑ CIP COL AZM
PaeAG1_01229	DR97_3944	–	15	1.3	1.45	Brown	–	Uncharacterized protein	–
PaeAG1_01591	rpoS (PA3622)	–	15	1.03	1.49	Turquoise	Transcription machinery	RNA polymerase sigma factor RpoS	↑ COL CIP ↓ AZM ↕ Cu
PaeAG1_01361	DR97_4078	–	19	-1.22	-1.48	Brown	–	Uncharacterized protein	–
PaeAG1_02250	rpmF (PA2970)	–	22	-1.17	-1.39	Brown	Ribosome	Ribosomal protein L32; Belongs to the bacterial ribosomal protein bL32 family	↓ CIP H_2_O_2_ ↕ COL Cu

*Cases with gray numbers refer to genes which were no selected as a DEG at that time (logFC and adjusted *p*-value).

**Cases with “-” refer to no annotation information.

***Results from other studies: ↑ up-regulated, ↓down-regulated, ↕ variable regulation or “– “ no information. All results from GEO-NCBI according to stress conditions: Cu (copper) from (Teitzel et al., 2006), CIP (ciprofloxacin) from (Cirz, O’Neill, Hammond, Head, & Romesberg, 2006), COL (colistin) from (Cummins, Reen, Baysse, Mooij, & O’Gara, 2009), AZM (Azithromycin) from (Kai et al., 2009) and H_2_O_2_ (hydrogen peroxide) from (Chang, Small, Toghrol, & Bentley, 2005).

Analysis of gene clusters of first-stage connected genes (Fig. 7B) showed not only the same profile of enriched pathways for those hub genes (Fig. 7A), but also other pathways such as lipids metabolism, phenazine biosynthesis, quorum sensing and others. These groups include many elements of phages, virulence factors and multiple uncharacterized genes, as well as one antibiotic resistance gene (PaeAG1_05751). The logFC values at time 5 h are shown in Figure S4C.

Six hub genes were consistently identified by both bottleneck and betweenness approaches (Table 4). Together with *rpoS* and *groL*, eight hub genes (57%) are part of the turquoise module, and all of them are up-regulated by CIP. All other genes are part of the brown (4) and blue modules (2). Only four genes were found to be down regulated, three of them belonging to the brown module.

To compare the expression profiles of hub genes to other studies, we included information in Table 4 of the effect of perturbations or stressors of *P. aeruginosa* in the modulation of gene expression. Similar effects of CIP on hub genes were found when comparing our results to a previous report^26^. The effects of azithromycin seem to be opposite to CIP for these genes. More variable results were found for other perturbations (e.g. colistin, copper and H_2_O_2_); and *lecB* was the only hub gene that was up-regulated for all perturbations.

Thus, as expected, hub genes are strongly linked to elements of highly connected gene clusters and at the same time with the key pathways in response to CIP. Together, these three elements (hub genes, gene clusters and enriched pathways) represent the determinants of the response to CIP in PaeAG1, many of them related to the bacterial growth modulation, as initially hypothesized.

### Concentration dependent effect of CIP in PaeAG1 phage induction

**Figure 8:**
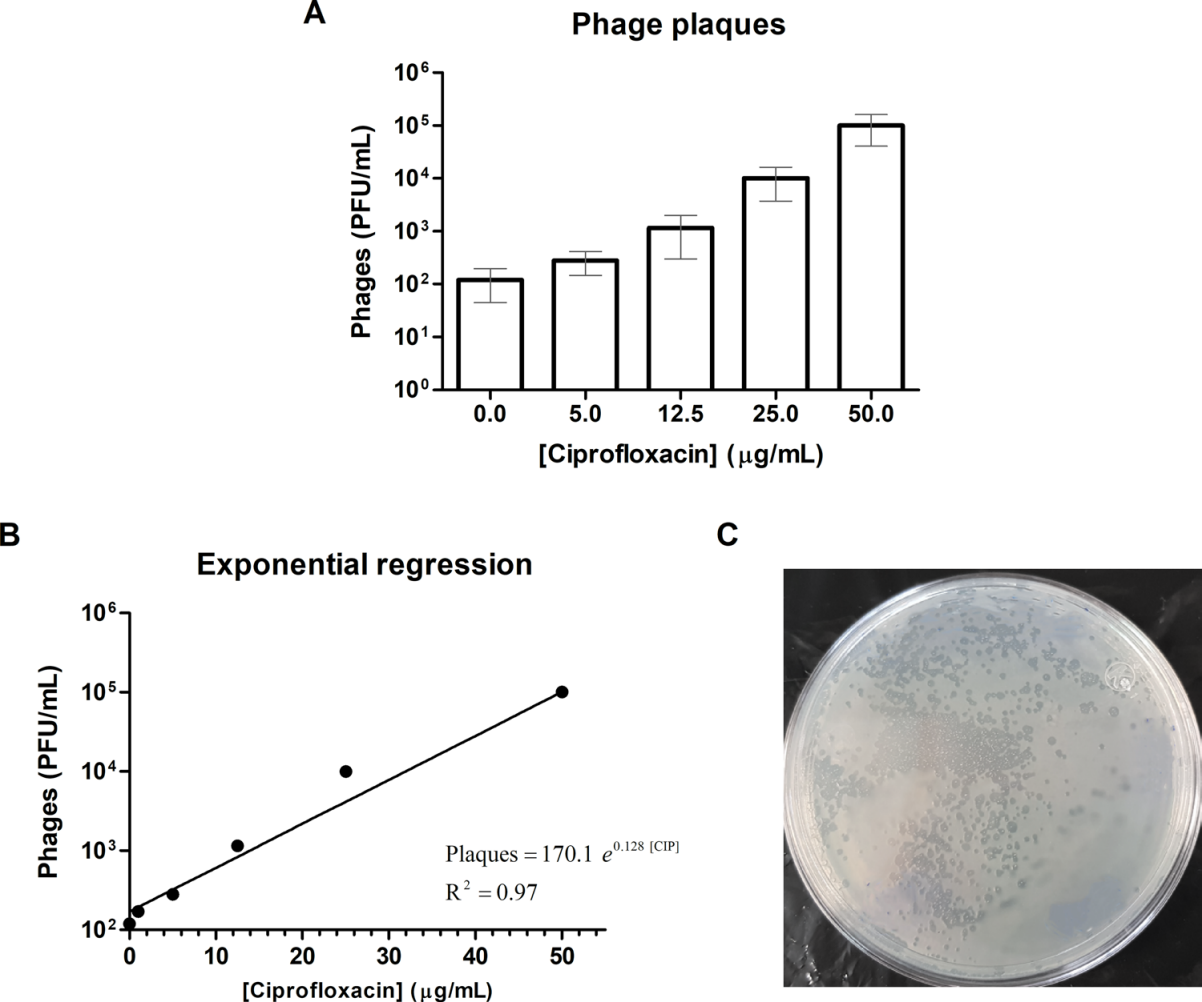
Phage plaques assay of PaeAG1 after exposure to ciprofloxacin. (**A**) Phages of PaeAG1 are induced under CIP exposure, with a pattern of higher induction of phage plaques at higher concentration of the drug, evidenced with an exponential regression as shown in (**B**). (**C**) Example of visualization of phage plaques on culture plate during in vitro assays.

According to transcriptomic analysis, phage genes were up-regulated under 12.5 μg/mL CIP treatment in PaeAG1. To validate these results at phenomic level, evaluation of lytic plaque formation was done using a phage plaque assay. As shown in Fig. 8A, after treatment with 12.5 µg/mL CIP, phage induction was increased by tenfold (1,000 PFU/mL) with respect to control condition without antibiotics, in concordance with the molecular findings. More drastic changes were evidenced for higher concentrations, where more than 10 000 or 100 000 PFU/mL were quantified for PaeAG1 after treatment with 25.0 and 50.0 µg/mL CIP concentrations, respectively. Figure 8C shows phage plaques on culture plate during in vitro assays. Unlike CIP, when the same analysis was done for imipenem and tobramycin (supplementary assay), no induction was evidenced. Indeed, a slight reduction was observed for imipenem (Supplementary Figure S1C–E, right).

Analysis of module genes to traits of PaeAG1 (phage production and time after CIP exposure) is presented in Fig. 5B. This analysis revealed a significant association of gene expression of the blue module, with changes at 2.5 h after CIP treatment and the low phage induction at this same time point. In a similar way, the turquoise module was significantly associated with changes of gene expression at 5 h and stronger phage induction. Other modules were not directly associated with these traits.

Altogether, these results indicate that phage induction in PaeAG1 is strongly dependent on CIP concentration, as shown with an exponential regression (R^2^ = 0.97) in Fig. 8B.

## Discussion

*P. aeruginosa* is a remarkable organism that can successfully resist, adapt, and survive in a wide variety of environments^29^. This versatility is conferred by the large proportion (> 8%) of regulatory genes encoded in its large genome (6–7.5 Mb, 7.2 Mb in the case of PaeAG1)^5,22^. This particular case of PaeAG1 strain is a high-risk ST-111 strain isolated from an immune-compromised patient in a Costa Rican Hospital, with resistance to multiple antibiotics including CIP and carbapenems. Although many *P. aeruginosa* strains are resistant to CIP^6,10,12,48^ and other antibiotics, the effects of sub-lethal concentrations on the development of antibiotic resistance had been ignored for decades due to the assumption that resistance emerges only with lethal concentrations (> MIC)^14^.

Therefore, we evaluated the effect of different CIP concentrations on PaeAG1 growth rate (Fig. 2). We detected a concentration-dependent reduction of growth rate as the CIP concentration was increased, similar to another study with CIP in *P. aeruginosa*^12^. We then employed RNA-Seq analysis to investigate the influence of a sub-inhibitory CIP concentration on the gene expression of PaeAG1 and its relationship with the bacterial growth, similar to recent studies in *P. aeruginosa*^76,77^ and other bacteria^16,44,78–81^. Differential expression analysis (Fig. 3) highlighted 518 DEGs at 2.5 and 5 h. Contrasting results have been previously reported in *P. aeruginosa* after CIP exposure, with some variations attributed mainly to differences in CIP concentration, time after exposure and/or the technical approach^12,26,48^.

We used a top-down systems biology approach to build the interaction network across the 518 DEGs. Interactions were modeled using a database-based method and co-expression analysis. A total of 14 hub genes, gene clusters and 15 KEGG pathways were associated with the molecular response to CIP, many of them related to bacterial growth, in line with other studies^26,82,83^. Discovery and description of these strong relationships between genes provided not only biological insights of the molecular regulation under stress conditions^42^, but also helped to reduce data complexity to only several central elements^40^, as other studies in *P. aeruginosa* PAO1^47^ and *E. coli*^40^.

### Sigma factor RpoS as a hub gene

Not surprisingly, one of the identified hub genes in PaeAG1 after CIP treatment was *rpoS*. This gene was only up-regulated at 5 h after exposure, suggesting a late regulation in comparison with other DEGs. RpoS is considered a master regulator of the general stress response^35^ which is induced when bacterial growth decreases, or under starvation, antibiotics and osmotic or oxidative stress^18^. In addition, RpoS participates in the protection of cellular macromolecules^18^, modulation of metabolism, virulence, and changes in cell envelope and morphology^11^. The overexpression of RpoS suggests that bacteria enter a stationary phase-like state upon stress conditions, as reported previously^44^. This is further supported by the observed significant lack of growth of bacteria under CIP treatment of various concentrations.

According to growth curves, PaeAG1 was in exponential phase at the time points used for the transcriptomic analysis (Fig. 2 and supplementary Figure S1A). This is a key point to ensure that RpoS induction (and all the response) is explained by the antibiotic and not due to stationary-phase entry (i.e. experimental design). The reliance of the observed changes on CIP treatment was further supported by the fact the curves at same conditions showed no changes for imipenem or tobramycin antibiotics (supplementary Figure S1C–E). Other fluoroquinolones were not tested for their effect on the production of phages in PaeAG1.

In addition, DNA binding site analysis using consensus sequence described in^75^ revealed 49 sites for RpoS in PaeAG1 genes, however none of these were found to be DEGs. In the same work, RpoS was regulating 772 genes at the stationary phase, of which 41 genes (5%) were identified as DEGs in our study. Since our analysis was performed at the exponential phase, the small number of common genes could be attributed to growth phase differences in each study. In another study using a de novo approach to identify binding sites using ChIP-Seq, RpoS showed to have 199 binding motifs in *P. aeruginosa* PA14^37^, including six transcription factors. In PaeAG1, 23 of these 199 genes corresponded to promoter regions of DEGs, including the RhlR and RpoS (itself) transcription factor genes. This suggests that 12% of the RpoS regulon was modulated by CIP in PaeAG1. Interestingly, context-centric analysis revealed that up to 28 transcription factors (including RpoS) are associated with the response to CIP, regulating gene expression with pleiotropic consequences and defining a crosstalk among factors in *P. aeruginosa*^37^.

On the other hand, the RpoS response contributes to the robustness of bacterial cells facing stress conditions, acting synergistically with the SOS response^18^. Although SOS response is known to be induced by CIP in *P. aeruginosa* and other bacteria^20,26,27,84^, in this study the SOS response was not significantly induced in response to CIP treatment at 2.5 and 5 h. The absence of SOS induction may be due to the timing and concentration of CIP treatment. In *E. coli,* dynamic models have shown that the time of response to cell stress is very fast, and stability of the SOS response can be achieved in minutes, around 30 min according to^85^ or up to 90 min according to^86^, until homeostasis is recovered or stronger stress responses are induced. Also, the SOS regulon of *P. aeruginosa* was established using a supra-inhibitory CIP concentration (8 × MIC) at times 30 and 120 min^26^. These differences in concentration and time (0.4 × MIC at 2.5 and 5 h for PaeAG1) could explain absence of SOS elements as DEGs. Our results are similar to another proteomic study using *P. aeruginosa*; profiles at 1.5, 5.5 and 14.5 h after CIP treatment were evaluated, and neither LexA nor other SOS proteins were differentially expressed, except for RecA, which was found to be up-regulated^87^.

### Phage induction as a response determinant

Regarding phage genes, two gene clusters with hub genes were defined in PaeAG1 after CIP treatment. Phage induction is known to be modulated upon stress conditions, including the SOS response^88^. As found recently for some antimicrobials, phage activity is product of pleiotropic regulation^89^. In the presence of sub-lethal concentrations of certain antibiotics, phages have been observed to be induced or to form larger phage plaques^88,90^. Under fluoroquinolones exposure, *P. aeruginosa* DNA is affected and the SOS response is triggered. In a similar manner to LexA, repressor cleavage reaction is stimulated by activated RecA, allowing virus assembly^91,92^, and killing of the bacterium^93^. In some cases, alternative RecA-independent mechanisms have been described^91,94^.

PaeAG1 has six prophages in the genome, including two complete elements^5^. After CIP exposure 85 phage genes were up-regulated, most of them from JBD44 (65 genes out of 105 JBD44 genes). In the co-expression analysis, when association between modules and traits was assessed, the turquoise module (Fig. 5) was significantly related to CIP exposure time and phage induction, indicating a coordinated gene expression activity belonging to this cluster/traits (Fig. 5B).

Although general information on PaeAG1 phages is scarce, there is evidence to suggest that JBD44 is one of the most prevalent in *P. aeruginosa*^95^. Effects of JBD44 induction on growth have been previously described in *P. aeruginosa* PAO1, showing that JBD44 expression significantly decreased the growth of PAO1, unlike other phages^96^. Similarly, SOS-mediated phage induction has been reported in *P. aeruginosa* PAO1^12,26^ and LESB58^97^. In addition, effect evaluation of several antibiotics found that CIP and norfloxacin (another fluoroquinolone) caused a high level of phage induction, but variable results were found for other antibiotics^92^. As observed in our experiments, no induction was found for imipenem nor tobramycin (supplementary Figure S1C–E).

The underlying relationship between the up-regulation of multiple phage genes in PaeAG1 after CIP exposure and the effect on bacterial lysis was validated through the effect of CIP concentrations in the phage induction. A concentration-dependent effect of CIP on both growth curves (rate reduction, Fig. 2) and phage plaques formation (exponential increment, Fig. 8) was demonstrated. This validated the transcriptomic findings of up-regulation of phage genes in PaeAG1.

In congruence with this and the enriched pathways in PaeAG1, it has been reported that cells can adapt to stresses by disrupting their own metabolism in such a way that will impair the success of phage activity^98^. This implies that effects are observed not only on the host cell fate but also modulation of different responses, including RpoS regulation. These changes can be a product of tight modulation of functions reliant on molecular interactions from both phage and bacteria^99^. Similarly, as phages generally appear to consume amino acid metabolites^100^, the bacterial up-regulation response of genes involved in amino acid catabolism has been suggested as a strategy for reducing the infection success^98^ and disrupting phage propagation^100^. Blasdel et al. 2017 found that *maiA, fahA, hmgA* and *hpd* genes of tyrosine catabolism were up-regulated by *P. aeruginosa* during phage activity^98^. In our study, all four genes were up-regulated, including *fahA* as a hub gene and a bottleneck element for the main phage gene cluster, indicating a catabolic effect after exposure to CIP that may be related to phage induction. More details of the *fahA* gene are discussed later.

Although different possibilities of the regulation of phage genes have been suggested, in the case of PaeAG1 phages, most of the predicted phage genes cannot be associated with a putative function, as in other studies^26^. This complicates the interpretation of the results for particular genes^99^. Validation of phage induction at phenomic level in congruence with transcriptomic results suggests that modulation of phages by CIP (but not for imipenem or tobramycin as discussed before) in PaeAG1 is possible. This is particularly relevant since this strain is a ST-111 high-risk clone and a critical organism Priority 1 (resistant to carbapenems) according to WHO^8^. Modulation could be achieve targeting phage production as a therapeutic option, with the advantage that the induced phages are resident elements of the genome and not exogenous elements as in other studies. Thus, treatment of antibiotic-resistant bacterial infections can potentially be improved by using phage therapy and traditional antibiotics, regardless if cells are growing in biofilms or as planktonic bacteria^88^. In addition, phage therapy can be used as a bactericidal element against multiresistant strains^93^. However, this does not necessarily apply to all *P. aeruginosa* strains since phage induction in other cases (with different strains and antibiotics) have been shown to be variable^92^.

### Other transcriptomic determinants

Of the 15 pathways recognized as enriched in PaeAG1 after CIP treatment, ribosomal activity, RNA degradation and several metabolic routes were prominently enriched with respect to others. Reduction in the abundance of ribosomal proteins and protein implicated in cell division over time indicate a shift by tolerant cells away from growth^87^, as it was evidenced by the changes in the growth curves under different CIP concentrations in PaeAG1. In the case of ribosomal activity, a cluster is clearly recognized in the whole network and the subnetwork of hub genes, where the *rpmF* gene is the up-regulated hub element. The *rpmF* gene encodes for the 50S ribosomal subunit protein L32, which is responsible for protein synthesis and membrane lipid synthesis^101^. It is also involved in multidrug tolerance by modulating biofilm formation and persister cell induction^102^.

Regarding metabolism, several reports have shown a down-regulation of energy production and carbohydrates, amino acids and lipids metabolism^15,36,87,103, 104^. Five hub genes (*sdhB, sdhC, prpC, acpP* and *fahA*) are particularly associated with metabolism. For instance, *fahA* is key in the inhibition of amino acid metabolism^105^*,* coding for a fumarylacetoacetase necessary for the tyrosine catabolism pathway. In addition, *fahA* is a topological bottleneck in the networks (Fig. 6A–C), separating the main phage genes cluster from the rest of the nodes. As detailed before, regulation of this gene could be used to restrict amino acids access to the phage and thus restraining the full phage activity^98^.

In the case of RNA degradation pathways, we identified groL (or groEL) as a hub gene, a homolog of heat shock protein 60^106^. DnaK and GroL are major ubiquitous chaperones that play crucial roles in promoting protein folding during normal growth and under stress conditions^107^ such as oxidative stress, antibiotics or heat^26,107,108^. In PaeAG1, both chaperones were up-regulated.

In relation to virulence factors, CIP modulated adherence and phenazines. A total of 19 DEGs implicated in adherence were identified with down-regulation observed for LPS O-antigen, flagella, and type IV pili biosynthesis elements. Similar results were found for *P. aeruginosa* after CIP treatment in another study^26^. Under other stress conditions, this down-regulation has been suggested to be a mechanism to avoid biofilm formation as a possible way to escape as planktonic cells^46^ and, in general, to modulate mechanisms for colonization, survival and invasion within the host tissues^93^.

Regarding phenazines, six genes were up-regulated. This profile is associated with tolerance to oxidative stress, iron availability, biofilms, virulence and killing microbial competitors^109^. Phenazine biosynthesis is regulated by the Rhl^76^ and PQS^110^ quorum sensing systems in *P. aeruginosa*. The *rhlR* gene was found to be up-regulated, suggesting a possible regulation of the phenazines.

More details of specific genes and their relationship with other virulence factors, antibiotic resistance and other responses (all with few number of DEGs) are discussed in the supplementary material “Extended discussion: Other transcriptomic determinants of PaeAG1 in response to CIP”.

**Figure 9:**
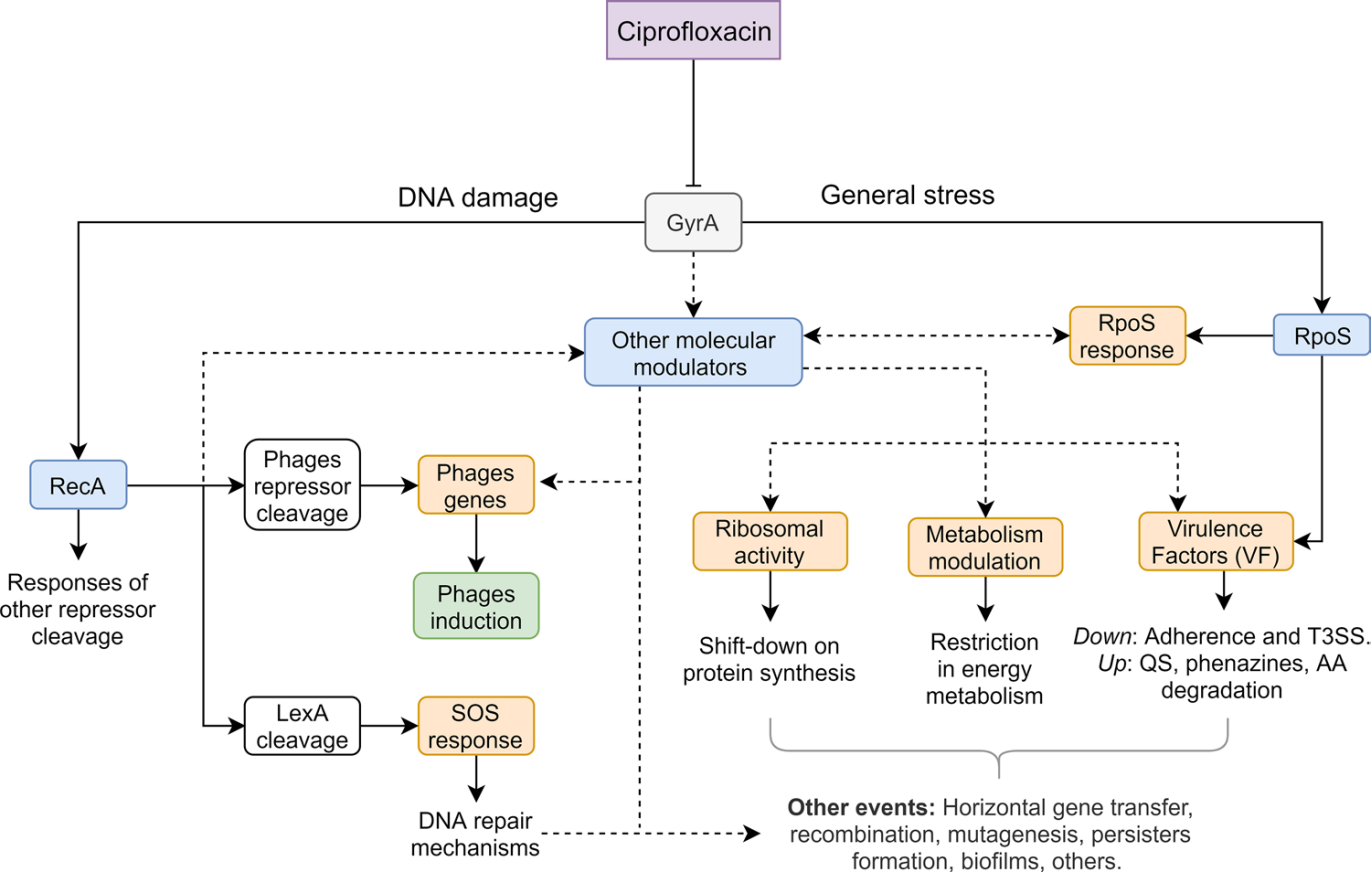
Conceptualization of effects of ciprofloxacin treatment in PaeAG1 at the molecular level. Effects of DNA damage triggers RecA increment, which cleaves different repressors such as LexA, inducing the SOS response, but also phages induction repressors, and other elements. The general stress induces the RpoS response, modulating different responses and virulence factors. Other modulators induce changes in the metabolic state of cells, expression of virulence factors, as well as the down-shift in ribosomal activity. Together, all changes imply modulation of multiple responses with pleiotropic effects at a molecular level and regulation of phenotypes to face the stress given by the antibiotic.

Altogether, the transcriptomic analysis in PaeAG1 allowed us to identify key molecular determinants of the response to CIP, many of them related to the bacterial grown, such as RpoS and phage induction. This agrees completely with our hypothesis in which transcriptomic response to CIP was related to bacterial growth modulation. After a DNA damage response is induced by sub-inhibitory CIP treatment, there is a subsequent pathway modulation and transcriptional changes that define changes in the bacterial growth. A conceptual representation of these results is shown in Fig. 9, aiming to integrate our results, literature reports and possible unknown connections.

All these features are particularly relevant for high-risk strains, such as PaeAG1. As it has been suggested, the biological markers of *P. aeruginosa* high-risk clones could be useful for the future design of specific treatments and infection control strategies^7^. Thus, more detailed analyses are needed to study the different levels of transcriptomic regulation in PaeAG1, including targeted expression analysis, other stress conditions, genetic and phenotypic variability, validation of the effect and power of hub genes, explorations of the relationship between presence of specific virulence traits and severity, and phage induction as a potential therapy.

## Conclusions

In this work, we report a concentration-dependent reduction of PaeAG1 growth rate upon increasing sub-inhibitory CIP concentrations by comparing growth curves. The RNA-Seq analysis of PaeAG1 after treatment with a sub-inhibitory CIP concentration allowed us to identify 518 DEGs along time at 2.5 and 5 h. Using a top-down systems biology approach, we identified diverse transcriptomic determinants: 14 hub genes, multiple gene clusters and 15 enriched pathways. These included down-regulation of pathways related to metabolism, ribosomal activity and adherence factors, most of them related to bacterial growth reduction. Phages, phenazines and specific virulence factors were found to be up-regulated. In most cases, hub genes and complex relationships were identified, showing pleiotropic effects that are mainly illustrated by clusters of highly connected genes. Two particular clusters of phages genes were up-regulated by CIP. Validation of CIP effects on phage induction was done at phenomic level with a phage plaque assay, showing an exponential induction as CIP was increased. To our knowledge, this is the first report of the analysis of CIP response in a ST-111 high-risk *P. aeruginosa* strain, in particular by a combined strategy using a top-down systems biology approach. This led us to identify transcriptomic determinants in response to CIP, including resident phages induction as a potential therapeutic strategy to overcome antibiotic resistance.

## Supplementary information

Supplementary Figure S1.

Supplementary Figure S2.

Supplementary Figure S3.

Supplementary Figure S4.

Supplementary Information 1.

Supplementary Information 2.

Supplementary Table S1.

Supplementary Table S2.

Supplementary Script 1.

Supplementary Script 2.

Supplementary Script 3.

## Data Availability

The RNA-seq raw data and processed files of transcripts quantification are available at the NCBI Gene Expression Omnibus (GEO) database under accession number GSE139866. Processed data and scripts for bioinformatics analyses (RNA-Seq data, differential expression using DESeq2 and co-expression analyses) are available at https://github.com/josemolina6/PaeAG1_CIP_RNA-Seq). Genome sequence and annotation files in all required formats for mapping and quality control of the RNA-Seq reads alignment are available from our previous work at https://github.com/josemolina6/PaeAG1_genome. More details of the genome assembly and annotation in^5^.

## Abbreviations

CIP: Ciprofloxacin
DEGs: Differentially expressed genes
GGI: Gene–gene interaction
GSEA: Gene set enrichment analysis
KEGG: Kyoto encyclopedia of genes and genomes
MIC: Minimum inhibitory concentration
MLST: Multilocus sequence typing
OD_600nm_: Optical density measured at 600 nm
PaeAG1: *Pseudomonas aeruginosa* Strain AG1
PCA: Principal components analysis
QC: Quality control
RNA-Seq: RNA sequencing
ST-111: Sequence type 111
WGCNA: Weighted gene co-expression network analysis
WHO: World Health Organization (WHO)
