# Supplementary figures and images for: Transcriptomic determinants of the response of ST-111 Pseudomonas aeruginosa AG1 to ciprofloxacin identified by a top-down systems biology approach
Source: Sci Rep. 2020 Aug 13;10:13717. doi: 10.1038/s41598-020-70581-2 (PMC7427096; doi:10.1038/s41598-020-70581-2)

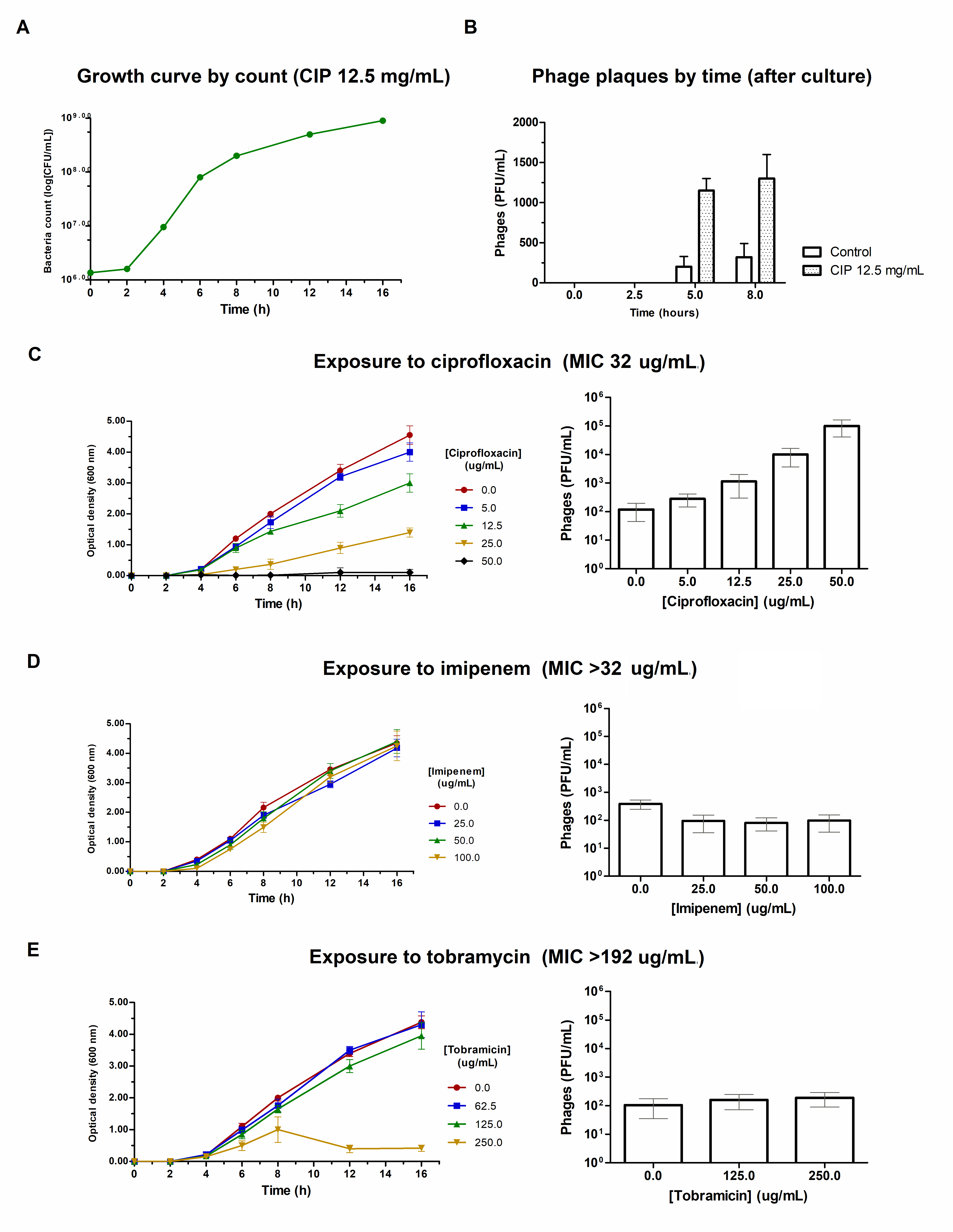

Supplement: Supplementary file 1 — Supplementary Figure S1. [file 41598_2020_70581_MOESM1_ESM.tif]
