# Supplementary material for: Transcriptomic determinants of the response of ST-111 Pseudomonas aeruginosa AG1 to ciprofloxacin identified by a top-down systems biology approach
Source: Sci Rep. 2020 Aug 13;10:13717. doi: 10.1038/s41598-020-70581-2 (PMC7427096; doi:10.1038/s41598-020-70581-2)
Supplement: Supplementary file 5 — Supplementary Information 1. [file 41598_2020_70581_MOESM5_ESM.docx]

**Extended discussion: Other transcriptomic determinants of PaeAG1 in response to CIP**

According to the different stages of the *P. aeruginosa* lifestyle between planktonic and sessile cells ^1^, the general profile of PaeAG1 after CIP exposure can be considered close to a planktonic cell where a non-active or survivor state enters to a stationary phase under stress conditions. This observation is congruent with a recent study in *P. aeruginosa* PAO1-UW, in which a transcriptomic analysis revealed that CIP treated cells had a similar profile to untreated biofilms forming cells (only 82 DEGs were identified), meanwhile more than 1700 genes were differentially expressed when treated cells were compared against to untreated planktonic bacteria ^2^.

*Other molecular responses*

Some stress responses mediate adaptation to conditions that cause protein misfolding in the periplasm, altered or impaired cell wall biogenesis, and cell wall remodeling ^3^. In congruence with this, another of the hub genes, *ygaU* (up-regulated at 2.5 h), is known to be directly regulated by RpoS in a cluster of genes related to general stress adaptation in *E. coli* ^4,5^. In *E. coli,* *ygaU* gene is related to bacterial envelope remodeling and is induced in response to a variety of stress conditions as well as alterations in cell wall composition ^3^.

On the other hand, LexA-DNA binding sites analysis found the 15 genes of the SOS response, *psrA* and *grpE,* and others. Although none of the SOS regulon genes were differentially expressed in PaeAG1, a possible LexA activity at initial times after CIP exposure can be suggested since *psrA* and *grpE* genes resulted up-regulated at 2.5 and 5 h, with effects on complementary responses to stress, as previously studied ^6,7^. In this context, PsrA regulates RpoS activity by binding to the promoter region of *rpos*, as previously described ^6^. In PaeAG1, both *rpoS* and *psrA* were found to be up-regulated under CIP exposure.

Regarding antibiotic resistance genes, only three genes were regulated by CIP in PaeAG1. The two genes of blaIMP-18 and blaOXA-2 lactamases (both in two independent integrons) were down-regulated. This contrasts with other studies were it has been shown that class I integron expression and recombination can be stimulated by antibiotic exposure ^8^. In addition, *ampC* (coding for another lactamase) was also down-regulated. AmpC overexpression has been shown in *P. aeruginosa* upon treatment with multiple antibiotics, including ceftazidime ^9^ and CIP ^10^. However, down regulation was shown using azithromycin ^11^ and copper ^12^, as in PaeAG1. AmpC is modulated by AmpR, a master regulator of gene expression in *P. aeruginosa,* which also modulates RpoS and multiple virulence factors ^13^.

Although our study is focused on the response to sub-inhibitory CIP concentration and not on specific antibiotic resistance mechanisms (including the effect of *gyrA* mutations), further transcriptomic analysis in PaeAG1 considering other CIP concentrations or interaction with other antibiotics, as well as comparison to other strains, could be used to identify particular patterns regarding resistance mechanisms. In this sense, an exhaustive transcriptome-wide association study found key genetic determinants of antibiotic resistance in 135 clinical *P. aeruginosa* isolates, including some particular patterns of resistance to fluoroquinolones and genomic data of gene expression and sequence variations in *gyrA* ^14^.

*Other virulence factors*

Virulence factors of *P. aeruginosa* include cell-associated elements such as
flagella, pili, lectins, alginate/biofilm, and lipopolysaccharide, as well as extracellular factors including proteases, cytotoxins, pyocyanin, siderophores, exotoxin A, and exoenzymes ^15^.

Two hub genes, *sdhB* and *sdhC,* are part of the succinate dehydrogenase *sdhABCD* operon, which encode enzymes that participate in aerobic and anaerobic metabolism, particularly in the tricarboxylic acid cycle ^16^. These genes were down-regulated in PaeAG1 after CIP exposure, similar to other studies under stress conditions ^10,12^. These *sdhABCD* genes are modulated negatively by *prrF* gene in *E. coli*, which contributes to iron homeostasis and also represses genes encoding enzymes for the degradation of anthranilate, a precursor of the *Pseudomonas* quinolone signal (PQS), one of the quorum sensing systems in *P. aeruginosa* ^17^. PQS is related to the expression of many virulence factors in *P. aeruginosa* ^17^, and its formation requires the up-regulation of *pqsABCDE* and *phnAB* operons ^18^ and metabolites including fatty acid metabolism and tricarboxylic acid cycle ^17^. In PaeAG1*, pqsB, pqsD, pqsE* and *phnB* genes were up regulated in both 2.5 and 5 h, suggesting activity in the modulation of virulence factors after CIP treatment. In the case of *prpC*, coding for citrate synthase 2, this is also involved in the tricarboxylic acid cycle, as well as it is a regulator of the T3SS ^19,20^. In PaeAG1 it was found to be up-regulated, as other reported cases ^10,21^.

For *acpP,* this hub gene codes for an acyl carrier protein (ACP) that plays a key role in fatty acid and complex lipid synthesis ^22^, proteins and non-ribosomal peptides synthesis, and as an inducer of quorum sensing ^22,23^. In addition, RpmF regulates several fatty acid biosynthetic genes such as *fabH, fabD* and *fabG,* all belonging to an operon related to the regulation of the synthesis of ribosomes, cell membranes and lipid biosynthesis ^24^*.* All three *rpmF, fabD* and *fabG* were down regulated in PaeAG1, suggesting a reduced protein and lipid synthesis after exposure to CIP (contrasting to phage proteins).

Another identified hub gene was the virulence factor *lecB*. This gene codes for lectin B and its expression is regulated by RpoS and quorum sensing systems in *P. aeruginosa* ^25^. The primary functional roles attributed to lectins is to mediate attachment to the host during infection, mediating adherence of *P. aeruginosa* to abiotic surfaces or target host cells ^26^. It binds to the outer membrane protein OprF during biofilm formation ^25^. Thus, LecB is a key adherence element that is involved in pathogenicity of *P. aeruginosa* by biofilm formation. After CIP treatment, *lecB* and *oprF* were found to be up-regulated in PaeAG1, as shown for other studies with CIP ^10^, colistin ^27^ and azithromycin ^11^.

Regarding quorum sensing, P. aeruginosa has four systems (Las, Rhl, PQS and IQS) ^15^. Although RpoS controls the synthesis of regulators of quorum sensing ^28^ after the general stress response induction ^29^ and these systems are well known to regulate the synthesis of many virulence factors ^30^, only one DEG was directly identified as part of the quorum sensing sytems, the *RhlR* gene (up-regulated). Other presumptive elements indirectly related to the PQS system were regulated by CIP, as discussed before, including the *pqsABCDE* and *phnAB* operons ^18^.

In relation to the five secretion systems of *P. aeruginosa*, only two genes were identified as DEGs, *pscE* and *yopB*. These two down-regulated genes belong to the T3SS ^31^. Construction of the T3SS apparatus is an energy-expensive process because it requires a large number of genes ^32^. Repression of the T3SS genes in response to DNA damage might allow cells to redirect energetic resources to the stress response ^33^. Regulation of this system is mediated by PrtR and PrtB ^34^, but these two genes were not differentially expressed in PaeAG1 after CIP exposure.

Although in other similar studies in *P. aeruginosa* PAO1 (using CIP) pyocines production was clearly induced ^35^, for PaeAG1 no specific pyocines were detected to be regulated. However, regulators including gene *PrtN* and neighbor genes of the same operon (PA0613 and PA0615) were up regulated, showing possible modulation of pyocines pathways in other levels.

Other representative virulence factors including LasA and LasB proteases, and hcnB toxin were found up-regulated with CIP, suggesting an increased capacity of *P. aeruginosa* to conquer environments in response to the stress state ^36^. LasA and LasB, jointly with exotoxin A and multiple proteases, are secreted by a type II secretion system or T2SS ^31^. Activity of T2SS is suggested although no specific gene of this system were identified as DEGs. Stress conditions can also up-regulate genes associated with iron homeostasis ^36,37^, including siderophores (such as pyoverdin and pyochelin) production to face iron limited environments ^31^. In PaeAG1 only one gene was found differentially expressed, the pyochelin *pchA* (up-regulated). Also, alginate biosynthesis has been associated with the response to oxidative and osmotic stress in *Pseudomonas* group ^38^, however in our case no DEGs were identified.

Altogether, the transcriptomic analysis in PaeAG1 indicate that CIP exposure can regulate key elements of the survival and pathogenesis of *P. aeruginosa.* Further analysis are required to detail the specific mechanisms and to eventually modulate the phenotypes and cell fate.

**References**

1. Coggan, K. A. & Wolfgang, M. C. Global regulatory pathways and cross-talk control pseudomonas aeruginosa environmental lifestyle and virulence phenotype. *Curr. Issues Mol. Biol.* **14,** 47–70 (2012).

2. Esani, S., Chen, T., Leung, K. P. & Van Laar, T. A. Transcriptome Sequence of Antibiotic-Treated Pseudomonas aeruginosa . *Microbiol. Resour. Announc.* **8,** 1–2 (2019).

3. Bernal-Cabas, M., Ayala, J. A. & Raivio, T. L. The Cpx envelope stress response modifies peptidoglycan cross-linking via the L,D-transpeptidase LdtD and the novel protein YgaU. *J. Bacteriol.* **197,** 603–14 (2015).

4. Metzner, M., Germer, J. & Hengge, R. Multiple stress signal integration in the regulation of the complex σS-dependent csiD-ygaF-gabDTP operon in Escherichia coli. *Mol. Microbiol.* **51,** 799–811 (2003).

5. Weber, H. *et al.* Genome-wide analysis of the general stress response network in Escherichia coli: sigmaS-dependent genes, promoters, and sigma factor selectivity. *Society* **187,** 1591–1603 (2005).

6. Kojic, M. & Venturi, V. Regulation of rpoS gene expression in Pseudomonas: involvement of a TetR family regulator. *J. Bacteriol.* **183,** 3712–20 (2001).

7. Sycheva, L. V., Permina, E. A. & Gelfand, M. S. Taxon-specific regulation of the SOS response in γ-proteobacteria. *Mol. Biol.* **41,** 827–835 (2007).

8. Andersson, D. I. & Hughes, D. Microbiological effects of sublethal levels of antibiotics. *Nat. Rev. Microbiol.* **12,** 465–478 (2014).

9. Cabot, G. *et al.* Evolution of Pseudomonas aeruginosa antimicrobial resistance and fitness under low and high mutation rates. *Antimicrob. Agents Chemother.* **60,** 1767–1778 (2016).

10. Cirz, R. T., O’Neill, B. M., Hammond, J. A., Head, S. R. & Romesberg, F. E. Defining the Pseudomonas aeruginosa SOS response and its role in the global response to the antibiotic ciprofloxacin. *J. Bacteriol.* **188,** 7101–7110 (2006).

11. Kai, T. *et al.* A low concentration of azithromycin inhibits the mRNA expression of N-acyl homoserine lactone synthesis enzymes, upstream of lasI or rhlI, in Pseudomonas aeruginosa. *Pulm. Pharmacol. Ther.* **22,** 483–486 (2009).

12. Teitzel, G. M. M. *et al.* Survival and growth in the presence of elevated copper: Transcriptional profiling of copper-stressed Pseudomonas aeruginosa. *J. Bacteriol.* **188,** 7242–7256 (2006).

13. Balasubramanian, D. *et al.* The regulatory repertoire of pseudomonas aeruginosa AmpC ß-lactamase regulator AmpR includes virulence genes. *PLoS One* **7,** (2012).

14. Khaledi, A. *et al.* Transcriptome profiling of antimicrobial resistance in Pseudomonas aeruginosa. *Antimicrob. Agents Chemother.* **60,** 4722–4733 (2016).

15. Lin, J., Cheng, J., Wang, Y. & Shen, X. The Pseudomonas Quinolone Signal (PQS): Not Just for Quorum Sensing Anymore. *Front. Cell. Infect. Microbiol.* **8,** 230 (2018).

16. Tata, M. *et al.* RNAseq based transcriptional profiling of Pseudomonas aeruginosa PA14 after shortand long-term anoxic cultivation in synthetic cystic fibrosis sputum medium. *PLoS One* **11,** 1–18 (2016).

17. Oglesby, A. G. *et al.* The influence of iron on Pseudomonas aeruginosa physiology: A regulatory link between iron and quorum sensing. *J. Biol. Chem.* **283,** 15558–15567 (2008).

18. Blasdel, B. G., Chevallereau, A., Monot, M., Lavigne, R. & Debarbieux, L. Comparative transcriptomics analyses reveal the conservation of an ancestral infectious strategy in two bacteriophage genera. *ISME J.* **11,** 1988–1996 (2017).

19. Tielen, P. *et al.* Regulatory and Metabolic Networks for the Adaptation of Pseudomonas aeruginosa Biofilms to Urinary Tract-Like Conditions. *PLoS One* **8,** e71845 (2013).

20. Dong, Y.-H., Zhang, X.-F. & Zhang, L.-H. The global regulator Crc plays a multifaceted role in modulation of type III secretion system in Pseudomonas aeruginosa. *Microbiologyopen* **2,** 161–72 (2013).

21. Chang, W., Small, D. A., Toghrol, F. & Bentley, W. E. Microarray analysis of Pseudomonas aeruginosa reveals induction of pyocin genes in response to hydrogen peroxide. *BMC Genomics* **6,** 1–14 (2005).

22. De Lay, N. R. & Cronan, J. E. Gene-specific random mutagenesis of Escherichia coli in vivo: isolation of temperature-sensitive mutations in the acyl carrier protein of fatty acid synthesis. *J. Bacteriol.* **188,** 287–96 (2006).

23. Kutchma, A. J., Hoang, T. T. & Schweizer, H. P. Characterization of a Pseudomonas aeruginosa fatty acid biosynthetic gene cluster: Purification of acyl carrier protein (ACP) and malonyl-coenzyme A:ACP transacylase (FabD). *J. Bacteriol.* **181,** 5498–5504 (1999).

24. Podkovyrov, S. & Larson, T. J. Lipid biosynthetic genes and a ribosomal protein gene are cotranscribed. *FEBS Lett.* **368,** 429–31 (1995).

25. Funken, H. *et al.* Specific Association of Lectin LecB with the Surface of Pseudomonas aeruginosa: Role of Outer Membrane Protein OprF. *PLoS One* **7,** 1–8 (2012).

26. Passos da Silva, D. *et al.* The Pseudomonas aeruginosa lectin LecB binds to the exopolysaccharide Psl and stabilizes the biofilm matrix. *Nat. Commun.* **10,** 1–11 (2019).

27. Cummins, J., Reen, F. J., Baysse, C., Mooij, M. J. & O’Gara, F. Subinhibitory concentrations of the cationic antimicrobial peptide colistin induce the pseudomonas quinolone signal in Pseudomonas aeruginosa. *Microbiology* **155,** 2826–2837 (2009).

28. Thi Bach Nguyen, H. *et al.* Negative Control of RpoS Synthesis by the sRNA ReaL in Pseudomonas aeruginosa. *Front. Microbiol.* **9,** 1–10 (2018).

29. Asfahl, K. L. & Schuster, M. Additive effects of quorum sensing anti-activators on Pseudomonas aeruginosa virulence traits and transcriptome. *Front. Microbiol.* **8,** (2018).

30. Suh, S. J. *et al.* Effect of rpoS mutation on the stress response and expression of virulence factors in Pseudomonas aeruginosa. *J. Bacteriol.* **181,** 3890–3897 (1999).

31. Chatterjee, M. *et al.* Antibiotic resistance in Pseudomonas aeruginosa and alternative therapeutic options. *Int. J. Med. Microbiol.* **306,** 48–58 (2016).

32. Wu, W. & Jin, S. PtrB of Pseudomonas aeruginosa suppresses the type III secretion system under the stress of DNA damage. *J. Bacteriol.* **187,** 6058–6068 (2005).

33. Yahr, T. L. & Wolfgang, M. C. Transcriptional regulation of the Pseudomonas aeruginosa type III secretion system. *Mol. Microbiol.* **62,** 631–640 (2006).

34. Sun, Z. *et al.* PrtR homeostasis contributes to Pseudomonas aeruginosa pathogenesis and resistance against Ciprofloxacin. *Infect. Immun.* **82,** 1638–1647 (2014).

35. Brazas, M. D., Brazas, M. D., Hancock, R. E. W. & Hancock, R. E. W. Ciprofloxacin Induction of a Susceptibility Determinant in Pseudomonas aeruginosa. *Antimicrob. Agents Chemother.* **49,** 3222–3227 (2005).

36. Gonzalez, M. R. *et al.* Transcriptome analysis of Pseudomonas aeruginosa cultured in human burn wound exudates. *Front. Cell. Infect. Microbiol.* **8,** 1–14 (2018).

37. Quintana, J., Novoa-Aponte, L. & Argüello, J. M. Copper homeostasis networks in the bacterium Pseudomonas aeruginosa. *J. Biol. Chem.* **292,** 15691–15704 (2017).

38. Lovelace, A. H., Smith, A. & Kvitko, B. H. Pattern-triggered immunity alters the transcriptional regulation of virulence-associated genes and induces the sulfur starvation response in pseudomonas syringae pv. tomato DC3000. *Mol. Plant-Microbe Interact.* **31,** 750–765 (2018).
