# Supplementary material for: Transcriptomic determinants of the response of ST-111 Pseudomonas aeruginosa AG1 to ciprofloxacin identified by a top-down systems biology approach
Source: Sci Rep. 2020 Aug 13;10:13717. doi: 10.1038/s41598-020-70581-2 (PMC7427096; doi:10.1038/s41598-020-70581-2)
Supplement: Supplementary file 6 — Supplementary Information 2. [file 41598_2020_70581_MOESM6_ESM.docx]

**Transcriptomic determinants of the response of ST-111 *Pseudomonas aeruginosa* AG1 to ciprofloxacin identified by a top-down**

**systems biology approach**

José Arturo Molina-Mora^1^*, Diana Chinchilla-Montero^1^, Maribel Chavarría-Azofeifa^1^, Alejandro J. Ulloa-Morales^2^, Rebeca Campos-Sánchez^3^, Rodrigo Mora-Rodríguez^1^, Leming Shi^4^ & Fernando García^1^

Centro de Investigación en Enfermedades Tropicales (CIET)^1^ and Centro de Investigación en Biología Celular y Molecular (CIBCM)^3^, Facultad de Microbiología, Universidad de Costa Rica, San José, Costa Rica.

Chemical Genomics Centre (CGC)^2^, Max-Planck-Institute for Molecular Physiology, Dortmund, Germany.

Human Phenome Institute (HuPI)^4^, Fudan University, Shanghai, China.

*Corresponding author: [jose.molinamora@ucr.ac.cr](mailto:jose.molinamora@ucr.ac.cr)

**SUPPLEMENTARY MATERIAL LEGENDS**

**Figure S1. Supplementary experimental assays to study PaeAG1 and exposure to ciprofloxacin.** (A) The growth curve of PaeAG1 exposed to CIP (12.5 ug/mL) was estimated using a standard plate count strategy (Colony Forming Units, CFU). (B) Evaluation of phage plaque induction after initial culture, under control or treatment conditions. (C), (D) and (E) correspond to the assessment of growth and phage induction of PaeAG1 after treatment with ciprofloxacin, imipenem and tobramycin, respectively.

**Figure S2. Evaluation of transcriptomic data of PaeAG1 exposed to Ciprofloxacin.** (A) Fitted curve to the dispersion estimates for each gene, as requirement to ensure data is a good fit for the DESeq2 model. (B) Dispersion of counts before and after normalization, making samples comparable. (C) PCA analysis shows similar profile of replicates in each condition at times 0, 2.5 and 5 hours, as expected. (D) Similar results are obtained using clustering analysis of samples.

**Figure S3. Gene-gene interaction (GGI) large scale network of differentially expressed genes in PaeAG1 after 2.5 and 5 h ciprofloxacin treatment, using a database-based method for prediction of interactions.** Increment from 248 to 284 nodes (DEGs) were triggered along the time, after 2.5 (A) and 5 (B) hours after exposure to ciprofloxacin. Lack of specific information in the database for PaeAG1 explains multiple not connected elements in the graph.

**Figure S4. Definitive large scale network of DEGs showing distribution of genes by modules and logFC values, after exposition of PaeAG1 to ciprofloxacin.** (A) Definitive network showing the distribution of co-expression modules. (B) Final network showing logFC for time 5 h. Gray nodes represent genes that were differentially expressed only at time 2.5 h (i.e no logFC value is displayed at time 5 h). (C) First-stage subnetwork of hub genes, showing logFC.

**Supplementary Text. Extended discussion: Other transcriptomic determinants of PaeAG1 in response to CIP.**

**Supplementary Table S1. DEGs and annotations.**

**Supplementary Table S2. Regulators of DEGs.**

**Supplementary_Script_1_RNASeq_QC_alignment**

**Supplementary_Script_2_Differential_expression_analysis**

**Supplementary_Script_3_co-expression analysis**
